# Exposure to Tick-Borne Pathogens in Cats and Dogs Infested With *Ixodes scapularis* in Quebec: An 8-Year Surveillance Study

**DOI:** 10.3389/fvets.2021.696815

**Published:** 2021-07-15

**Authors:** Lauriane Duplaix, Victoria Wagner, Salima Gasmi, L. Robbin Lindsay, Antonia Dibernardo, Karine Thivierge, Christopher Fernandez-Prada, Julie Arsenault

**Affiliations:** ^1^Department of Pathology and Microbiology, Faculty of Veterinary Medicine, Université de Montréal, Saint-Hyacinthe, QC, Canada; ^2^Groupe de Recherche en Épidémiologie des Zoonoses et Santé Publique, Faculty of Veterinary Medicine, Université de Montréal, Saint-Hyacinthe, QC, Canada; ^3^Groupe de Recherche sur les Maladies Infectieuses des Animaux de Production, Université de Montréal, Saint-Hyacinthe, QC, Canada; ^4^Policy Integration and Zoonoses Division, Centre for Food-borne, Environmental and Zoonotic Infectious Diseases, Public Health Agency of Canada, Saint-Hyacinthe, QC, Canada; ^5^Zoonotic Diseases and Special Pathogens Division, National Microbiology Laboratory, Public Health Agency of Canada, Winnipeg, MB, Canada; ^6^Laboratoire de Santé Publique du Québec, Institut National de Santé Publique du Québec, Sainte-Anne-de-Bellevue, QC, Canada; ^7^Institute of Parasitology, Faculty of Agricultural and Environmental Sciences, McGill University, Macdonald Campus, Sainte-Anne-de-Bellevue, QC, Canada; ^8^Department of Microbiology and Immunology, Faculty of Medicine, McGill University, Montreal, QC, Canada

**Keywords:** *Anaplasma phagocytophilum*, *Babesia microti*, *Borrelia burgdorferi*, cat, dog, *Ixodes scapularis*, vector-borne, zoonosis

## Abstract

Cats that spend time outdoors and dogs are particularly at risk of exposure to ticks and the pathogens they transmit. A retrospective study on data collected through passive tick surveillance was conducted to estimate the risk of exposure to tick-borne pathogens in cats and dogs bitten by blacklegged ticks (*Ixodes scapularis*) in the province of Quebec, Canada, from 2010 to 2017. Blacklegged ticks collected from these host animals were tested by PCR for *Borrelia burgdorferi* sensu stricto, *Borrelia miyamotoi, Anaplasma phagocytophilum*, and *Babesia microti*. A total of 13,733 blacklegged ticks were collected from 12,547 animals. Most ticks were adult females and partially engorged. In total, 1,774 cats were infested with ticks and 22.6 and 2.7% of these animals were bitten by at least one tick infected with *B. burgdorferi* and *A. phagocytophilum*, respectively. For the 10,773 tick infested dogs, 18.4% were exposed to *B. burgdorferi* positive ticks while 1.9% of infested dogs were exposed to ticks infected with *A. phagocytophilum*. The risk of exposure of both cats and dogs to *B. miyamotoi* and *B. microti* was lower since only 1.2 and 0.1% of ticks removed were infected with these pathogens, respectively. Traveling outside of the province of Quebec prior to tick collection was significantly associated with exposure to at least one positive tick for *B. burgdorferi, A. phagocytophilum* and *B. microti*. Animals exposed to *B. burgdorferi* or *B. miyamotoi* positive tick(s) were at higher risk of being concurrently exposed to *A. phagocytophilum;* higher risk of exposure to *B. microti* was also observed in animals concurrently exposed to *B. burgdorferi*. The odds of dogs having *B. burgdorferi* antibodies were higher when multiple ticks were collected on an animal. The testing and treatment strategies used on dogs bitten by infected ticks were diverse, and misconceptions among veterinarians regarding the treatment of asymptomatic but *B. burgdorferi*-seropositive dogs were noted. In conclusion, our study demonstrates that cats and dogs throughout Quebec are exposed to blacklegged ticks infected with *B. burgdorferi* and *A. phagocytophilum*, and veterinarians across the province need to be aware of this potential threat to the health of pets and their owners.

## Introduction

Given their habits, dogs and cats that spend time outdoors are particularly at risk of contracting tick-borne diseases (1, 2). In parts of eastern and central North America, blacklegged ticks, *Ixodes scapularis*, are the primary vector for the spirochete *Borrelia burgdorferi* sensu stricto (hereafter referred to as *B. burgdorferi*), which is the causative agent of Lyme disease (LD) (3). Infection with *B. burgdorferi* in dogs is most often asymptomatic, but may lead to febrile illness, inappetence, and arthropathy in some animals (4). Neurological signs have also been reported in dogs but are not well-understood, and neither is the fatal myocardial condition that has been documented in Boxer puppies (1). Other possible sequelae of *B. burgdorferi* infection include the ever-elusive Lyme nephritis, a protein-losing nephropathy, which is a dangerous condition that occurs in <1–2% of *B. burgdorferi* seropositive dogs (5). As erythema migrans is not known to occur in dogs (2), many canine cases of LD are overlooked until the onset of arthritis or nephritis (1, 6–8). In contrast, cats rarely develop clinical LD following natural infection (9, 10).

Blacklegged ticks can also transmit *Anaplasma phagocytophilum, Babesia microti* and *Borrelia miyamotoi*. *A. phagocytophilum* causes granulocytic anaplasmosis. Much like *B. burgdorferi* infections, most cases of granulocytic anaplasmosis in dogs are asymptomatic, although some animals may display non-specific signs including: fever, lethargy, lameness, hemolytic anemia, and thrombocytopenia (11–15). Although clinical disease as a result of exposure of cats to *A. phagocytophilum* has been reported, most feline exposures to infected ticks result in asymptomatic infection. The high seroprevalence of antibodies against *A. phagocytophilum* in healthy cats is further evidence that infections usually do not result in disease (16–18). *Babesia microti* causes a malaria-like illness in humans, but it is uncertain whether it can cause disease in companion animals and its taxonomic status is highly debated (19, 20). Nevertheless, this parasite may occasionally cause hematological abnormalities, azotemia, and death in dogs, but tends not to cause clinical signs in cats (21–23). In contrast, *B. miyamotoi* is not known to cause illness in dogs or cats (24–26).

Many of the aforementioned tick-borne diseases were long considered of low risk to Canadians and their pets given that blacklegged tick populations in North America were mostly limited to the United States (27). In recent years, however, this vector has extended its range to become prevalent in eastern and central portions of Canada, which led to an increased incidence of LD in humans and to serological evidence of exposure to *A. phagocytophilum* in humans and dogs (28, 29). Seroprevalence studies in dogs also suggest that the risk of exposure to *B. burgdorferi* has been increasing in Canada in recent years (29, 30).

Management of LD and granulocytic anaplasmosis in dogs is complicated and may vary greatly between veterinary practitioners. Currently, serology is the recommended method for detection of exposure to *B. burgdorferi*, using one of the existing commercial or reference laboratory tests (1). However, no consensus has been reached by the American College of Veterinary Internal Medicine (ACVIM) on whether asymptomatic *B. burgdorferi* seropositive dogs should be treated—though there is agreement that animals testing seropositive should be monitored for proteinuria (1, 4). Of note, although serological testing is considered useful evidence of exposure to *B. burgdorferi*, there is little evidence that antibody titers can reliably predict the onset of clinical signs (1). In dogs suffering from Lyme arthritis, a 4-week course of antibiotics is recommended with doxycycline as first-line choice (1). For granulocytic anaplasmosis, screening for infection by examining stained blood smears under the microscope is common, and diagnosis can be confirmed by detection of serum antibody or PCR on whole blood samples (31, 32). Doxycycline is generally the recommended course of treatment for clinical anaplasmosis, as with LD (31, 32). Tick control is advised for all at-risk dogs and cats for the prevention of tick-borne infections (1). Vaccines for *B. burgdorferi* are also available, but no consensus was reached among panelists of the ACVIM on their use in endemic areas (1). More research and veterinarian education to demystify the protocols for prevention and management of suspected cases of tick-borne disease are imperative as these diseases become more prevalent in Canada.

This study has several objectives, but the overarching aim is to provide crucial insight into the risk of exposure of companion animals in Quebec, Canada to tick-borne pathogens. Risk of exposure was estimated using *I. scapularis* ticks collected from cats and dogs and submitted to the Laboratoire de santé publique du Québec (LSPQ) from 2010 to 2017. Blacklegged ticks were tested for pathogens using PCR to (1) estimate the risk of exposure to ticks infected with *B. burgdorferi, A. phagocytophilum, B. miyamotoi*, or *B. microti* in cats and dogs bitten by at least one blacklegged tick (by year, administrative region, animal species (cat or dog) and based on the host's history of travel); (2) determine the presence of spatiotemporal clusters of infested cats and dogs exposed to pathogen infected ticks (by pathogen type); (3) assess the risk factors for exposure to infected ticks (by pathogen type) in infested animals; (4) determine the probability of coexposure to multiple pathogens from positive ticks; and (5) describe veterinarians' current practices for the management of animals bitten by ticks with known exposure to infected ticks. This unique bank of information provides a portrait of the recent evolution of tick-borne pathogens in ticks collected from cats and dogs in Quebec, as well as highlighting the management strategies implemented by veterinarians. As a whole, this study will contribute to the development of optimal prevention and management strategies as tick-borne infections become more prevalent in the province of Quebec.

## Materials and Methods

### Tick Collection and Laboratory Analyses

A passive surveillance program for blacklegged ticks has been ongoing in the province of Quebec since 1990. In the veterinary component of this surveillance system, ticks collected from companion animals (mostly cats and dogs) were submitted by participating veterinary clinics to the LSPQ. More than 500 veterinary clinics have participated in this surveillance program over time. All ticks received by the LSPQ were submitted for species identification and developmental stage evaluation following standard keys and taxonomic references (33–37). Three levels of engorgement were determined visually using semi-quantitative scores for most of the tick species and stages: not engorged, partially engorged and fully engorged. For *I. scapularis* females, specific size ranges were used to determine the level of engorgement: not engorged: <3.7 mm, partially engorged: 3.7 to 8 mm, fully engorged: >8 mm. For every tick received at the LSPQ, the date of tick collection, the travel history of the animal (with departure and return dates), as well as information on tick instars (larvae, nymph, adult male or adult female), tick condition (dead or alive), and degree of engorgement were compiled.

As part of the surveillance program, all ticks submitted to the LSPQ and identified as *I. scapularis* were then sent to the National Microbiology Laboratory (NML) of the Public Health Agency of Canada for polymerase chain reaction (PCR) testing. The NML routinely tests blacklegged ticks, submitted through passive tick surveillance, for the human pathogens *B*. *burgdorferi, B*. *miyamotoi, A*. *phagocytophilum*, and *B*. *microti* by real-time PCR (RT-PCR), as previously described (38, 39). Briefly, QIAGEN DNeasy 96 tissue kits (QIAGEN Inc., Mississauga, ON) were used according to the manufacturer's protocol for DNA extraction. A duplex RT-PCR assay was employed to screen samples for *Borrelia* spp. (including all members of the *B. burgdorferi* sensu lato group) and *A*. *phagocytophilum* by targeting the 23S rRNA and *msp2* genes, respectively (39). Analysis for *B*. *microti* was conducted using the methods described by Nakajima et al. for the detection of the *CCT eta* gene (40), followed by an in-house RT-PCR assay targeting the 18S rRNA gene on positive samples. Subsequently, all *Borrelia* spp.-positive samples collected in 2014 were tested for *B*. *burgdorferi* using a confirmatory *ospA* RT-PCR assay, and for *B*. *miyamotoi* using an *IGS* real-time PCR assay. *B*. *miyamotoi-*positives were further verified using the *glpQ* RT-PCR assay (41). From 2014 onwards, *Borrelia* spp.-positive samples were confirmed by *ospA* and *glpQ* assays only. Samples were tested for *A*. *phagocytophilum* and *B*. *burgdorferi* throughout the study period, and PCR testing for *B. microti* and *B. miyamotoi* was added starting in 2013 and 2014, respectively. Most tick specimens were extracted and tested individually and most partially and fully engorged specimens were cut in half longitudinally, with DNA extracted from only one half of each specimen. If multiple ticks were collected from an animal, a pool of these ticks was tested by PCR.

In this study, data on blacklegged ticks collected from cats and dogs and submitted by veterinary clinics within the province of Quebec between 2010 and 2017, as well as the information gathered about these animals (see Section 2.6) were analyzed. All regions of Quebec were included, except for the administrative region of Montérégie. This region was excluded because, as of 2009, submissions from participating veterinary clinics located in this region were no longer accepted due to resource management issues. Also, data obtained from cats and dogs living in the Montérégie region from which ticks were collected in a clinic located in another region were excluded from our dataset since it would only represent partial data from this area. Similarly, data obtained from cats and dogs living outside the province of Quebec was also excluded.

### Prevalence of Exposure of Hosts to Infected Ticks

The risk of exposure to at least one blacklegged tick infected with *B. burgdorferi, A. phagocytophilum, B. microti*, or *B. miyamotoi* for tick infested cats and dogs residing in the province of Quebec was estimated with 95% exact confidence intervals (CI) by year, administrative region, and according to the host animal's history of travel 14 days prior to tick collection. Risk of pathogen exposure in tick infested cats and dogs was also described according to the month of tick collection, aggregated overall years of the study.

### Risk Mapping and Spatiotemporal Clusters of Exposure to Infected Ticks

**Figure 1:**
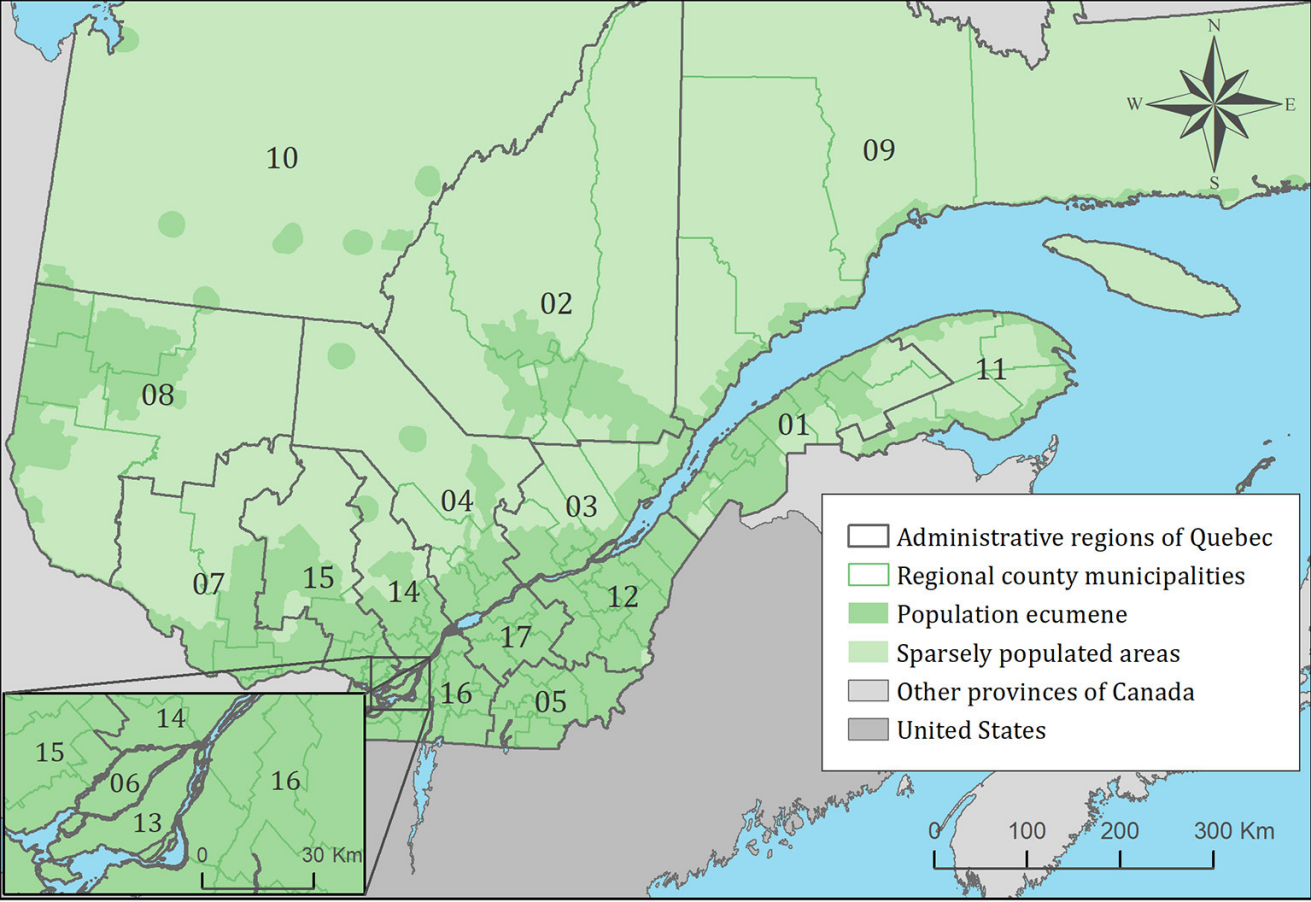
Administrative regions, regional county municipalities, population ecumene (i.e., land with population density ≥0.4 persons per km^2^) and sparsely populated areas (i.e., unpopulated land or land with population density <0.4 persons per km^2^) of the province of Quebec, Canada. Administrative regions are: 01: Bas-Saint-Laurent, 02: Saguenay-Lac-Saint-Jean, 03: Capitale-Nationale, 04: Mauricie, 05: Estrie, 06: Montréal, 07: Outaouais, 08: Abitibi-Témiscamingue, 09: Côte-Nord, 10: Nord-du-Québec, 11: Gaspésie-Îles-de-la-Madeleine, 12: Chaudière-Appalaches, 13: Laval, 14: Lanaudière, 15: Laurentides, 16: Montérégie, 17: Centre-du-Québec.

All cats and dogs were first geolocated at their owner's municipality of residence and were then aggregated at the regional county municipality for mapping (Figure 1). Choropleth maps were used to illustrate the spatial distribution of cats and dogs exposed to PCR-positive ticks among all infested animals overall years. All mapping was performed in ArcGIS 10.5.1 (ESRI, Redlands, CA, USA) using geographic boundary files from the 2016 Statistics Canada census for administrative regions and population ecumene. All mapping was performed by animal species (cats, dogs) and for the two most frequent pathogens (*B. burgdorferi, A. phagocytophilum*), and was limited to animals that did not travel outside their municipality of residence within 14 days of tick collection.

The spatiotemporal trends of the animal risk of exposure to at least one tick infected with *B. burgdorferi* and *A. phagocytophilum* was investigated using the Kulldorff scan test, performed separately for each animal species (cats, dogs) and pathogen. A Bernoulli model was used, scanning for the presence of spatiotemporal high-risk clusters of exposition to PCR-positive ticks, performed in SaTScan software version 9.6 (MA, USA) (42). Cases were defined as an animal bitten by at least one infected tick, and controls as an animal bitten by a tick or multiple ticks in which the pathogens of interest were not detected (i.e., negative controls). For this analysis, the spatial unit was the regional county municipality, and the temporal unit was the year (i.e., all cases and controls occurring within the same year and municipality were aggregated). Only cats and dogs that did not travel within 14 days of tick collection were used in this analysis. Statistical significance (*p-*value < 0.05) was determined based on 9999 Monte Carlo permutations.

### Factors Associated With Exposure to Infected Ticks

Logistic regressions were used to model animal exposure to infected ticks for each pathogen based on the maximum level of engorgement of ticks collected from an animal (i.e., in the following categories: fully engorged, partially engorged, or not engorged), number of ticks pooled for PCR testing (one tick vs. two or more ticks), animal species (cat vs. dog), month of tick collection (i.e., in the following categories: January-March, April-June, July-September, October-December) and maximum distance animals traveled in the 14 days prior to tick collection (i.e., in the following categories: no travel, out of municipality, out of administrative region, out of province). The condition (at least one tick alive vs. all dead ticks) and the quality (at least one intact vs. all damaged) of the submitted ticks were also included as control variables and to assess the potential impact of these variables on the likelihood of pathogen detection in a surveillance context. For animals from which more than one tick was collected, the best condition (alive), the best quality (intact) and the maximum level of engorgement (fully engorged) were used for data description and analyses for all specimens. Variables with a *p-*value <0.20 in the univariable logistic regression models were retained for modeling. Selected variables were included in a full multivariable model and submitted to a manual backward selection procedure using a *p-*value >0.05 as criteria for rejection. However, for *B. miyamotoi* and *B. microti*, due to the limited number of animals exposed to ticks infected with these pathogens, only univariable exact logistic regressions were done. Odds ratios with 95% CI were used to present the results. Statistical analyses were performed on SAS version 9.4 (SAS Institute Inc., Cary, NC, USA).

### Coexposure to Multiple Tick-Borne Pathogens

Coexposures of cats and dogs to the aforementioned agents through ticks were described, defined as an animal exposed to ticks coinfected with multiple pathogens or as an animal concurrently infested with multiple ticks infected with different pathogens. For each pairwise combination of pathogens, exact Chi-square tests were performed to evaluate if exposure to one pathogen increases the risk of being concurrently exposed to another one. Due to the limited number of coexposed hosts, cats and dogs were combined for these analyses.

### Description of Veterinarian Management Practices and Serological Outcome

When ticks tested positive for *B. burgdorferi* and/or *A. phagocytophilum* by PCR, a questionnaire was sent to the veterinary clinics by the LSPQ to collect information on the animal's clinical manifestations and case management, including diagnostic testing and results, treatment, and vaccination. This questionnaire was usually sent 6 to 12 weeks after reception of the tick for identification at the LSPQ, and was completed on a voluntary basis by a veterinarian or animal health technician. Based on data collected from this questionnaire, the diagnostic procedures performed by a veterinarian for animals bitten by *B. burgdorferi*- and/or *A. phagocytophilum*-infected tick(s) were described. For dogs on which a SNAP 4DX was performed at least 4 weeks after tick collection and/or a Lyme quantitative C_6_ antibody assay was performed at least 3 weeks after tick collection, the prevalence of seropositive dogs was calculated. Logistic regression models were used to explore the impact of the level of engorgement (or maximum level of engorgement among ticks collected from an animal and pooled for testing) and the number of ticks collected from one dog on the probability that a dog bitten by at least one *B. burgdorferi*-infected tick tested seropositive for this agent. The model-building approach was the same as previously described. Finally, the treatment and vaccination of these dogs were described.

## Results

### Description of Ticks Collected

A total of 13,733 blacklegged ticks were collected from cats and dogs by participating veterinary clinics during the study. In total, 13,445 were adult females, 243 were adult males, 41 were nymphs, and four were larvae. Condition and quality of ticks upon receipt at the LSPQ was available for 7,755 and 7,754 ticks, respectively as these statistics were compiled from April 2014 to 2017. Only 5% (*n* = 387) of these ticks were alive but most specimens were intact (77%; *n* = 5,973). Level of engorgement was available for all years of the surveillance program and for 13,669 ticks: 23% (*n* = 3,134) were fully engorged, 73% (*n* = 10,040) were partially engorged, and 4% (*n* = 495) were not engorged.

### Description of Animals Infested With Ticks

At least one blacklegged tick was collected from 12,547 cats or dogs over the course of this study. Information on the condition and quality of submitted ticks was available for 7,257 infested hosts, while level of tick engorgement was available for 11,026 infested animals. Among cats and dogs, 384 (5%) were bitten by at least one tick that was submitted alive and 5,547 (76.4%) were bitten by at least one tick that was submitted intact. In addition, 2,567 (23.3%), 8,084 (73.3%), and 375 (3.4%) of infested cats and dogs were bitten by at least one tick that was fully engorged, partially engorged, and not engorged, respectively.

**Figure 2:**
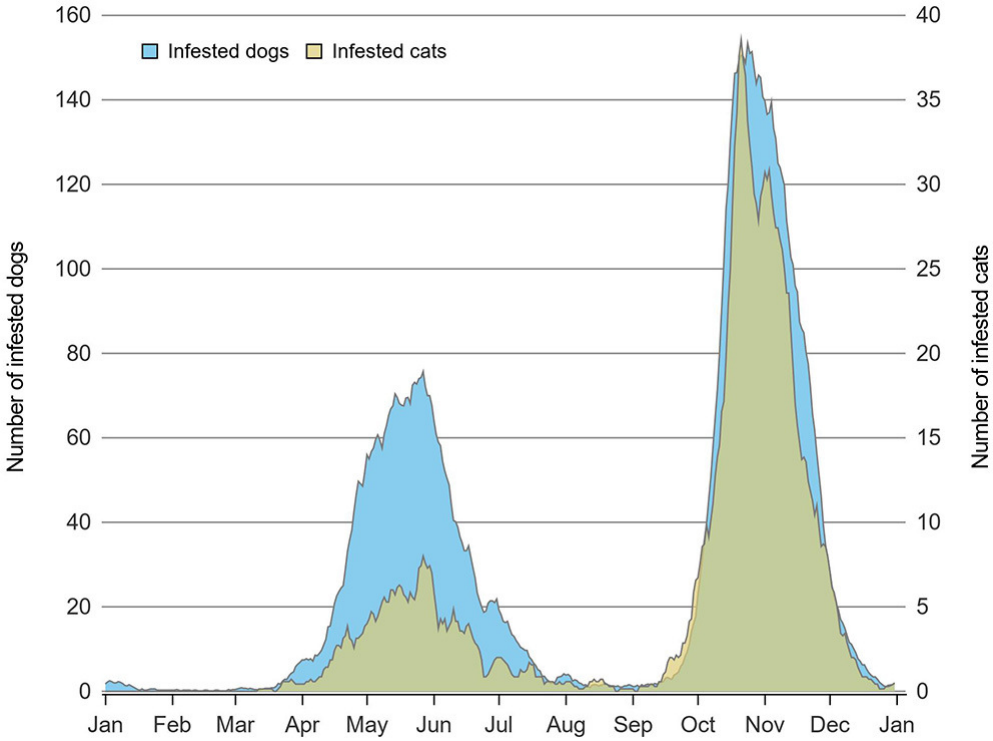
Daily number of dogs (*n* = 10,773) and cats (*n* = 1,774) infested with at least one adult tick in Quebec, Canada using a 7-day moving average on data from 2010 to 2017.

The number of tick-infested cats reported to the surveillance program increased from 122 in 2010 to 438 in 2017 and overall, 1,774 cats were infested with 1,905 blacklegged ticks. As for dogs, 657 were tick-infested in 2010 and the reported number of infested dogs increased to 2,296 in 2017 with an overall total of 10,773 dogs infested with 11,828 ticks. Multiple tick specimens were collected from 5.2% (*n* = 92) of infested cats and 5.0% (*n* = 535) of infested dogs. Among animals infested by multiple ticks, the median number of ticks collected and pooled for testing was two in the 92 cats (minimum = 2 ticks, maximum = 12 ticks) and also two in the 535 dogs (minimum = 2 ticks, maximum = 25 ticks). Cats were most often infested with individual adult (*n* = 1,666 instances) or nymphal (*n* = 16) ticks, although multiple adult and nymphal ticks were detected on 91 cats and one cat, respectively. Similarly, of the 10,773 infested dogs, 10,223 had only one adult tick, 16 had only one nymphal tick, 530 dogs were infested with multiple adult ticks, four were infested with both adult and nymphal ticks, and one dog was infested with all tick stages. The seasonal distribution of blacklegged tick infestation of cats and dogs is presented in Figure 2. A bimodal distribution pattern of infestation was noted for both cats and dogs; however, fewer cats were infested during the spring peak compared to dogs. In contrast, 34 of 37 animals infested with nymphal ticks were observed between May and August inclusively.

### Prevalence of Exposure of Hosts to Infected Ticks

The proportion of cats infested with *B. burgdorferi*-infected blacklegged ticks varied from 14.8% in 2010 to 25.3% in 2017 with the highest prevalence (25.4%) observed in 2014. For infested dogs, the proportion of dogs infested with *B. burgdorferi*-infected ticks ranged from 16.6% in 2010 to 19.4% in 2017 (Table 1). The proportion of cats and dogs exposed to *B. burgdorferi*-infected ticks also varied within the administrative region of residence. For example, as few as 4.2% of infested cats in Abitibi-Témiscamingue to 38.5% of cats from Saguenay-Lac-Saint-Jean were exposed to *B. burgdorferi*-infected ticks. For dogs, the regional risk of exposure to infected ticks varied from 0% in Nord-du-Québec to 25.5% in Côte-Nord (Table 1). Of note, *B. burgdorferi* was only detected in adult ticks or in pools of ticks including at least one adult. The monthly proportions of cats and dogs infested with *B. burgdorferi*-infected ticks is presented in Supplementary Table 1.

**Table 1:** Estimated risk of exposure to blacklegged ticks infected with *Borrelia burgdorferi* in infested cats and dogs per year and administrative region in Quebec, Canada from 2010 to 2017.

**Year and administrative region**	**Infested cats**				**Infested dogs**			
	**Total number**	**Number exposed to infected ticks**	**Risk of exposure**		**Total number**	**Number exposed to infected ticks**	**Risk of exposure**	
			**%**	**CI (95%)^a^**			**%**	**CI (95%)^a^**
**Year**								
2010	122	18	14.8	9.0–22.3	657	109	16.6	13.8-19.7
2011	266	58	21.8	17.0–27.3	1,207	218	18.1	15.9–20.4
2012	133	21	15.8	10.1–23.1	919	162	17.6	15.2–20.3
2013	213	48	22.5	17.1–28.7	1,271	228	17.9	15.9–20.2
2014	181	46	25.4	19.3–32.4	1,422	269	18.9	16.9–21.1
2015	192	42	21.9	16.2–28.4	1,371	257	18.8	16.7–20.9
2016	229	57	24.9	19.4–31.0	1,630	290	17.8	16.0–19.7
2017	438	111	25.3	21.3–29.7	2,296	445	19.4	17.8–21.1
Global	1,774	401	22.6	20.7–24.6	10,773	1978	18.4	17.6–19.1
**Administrative region**								
01	70	21	30.0	19.6–42.1	185	44	23.8	17.8–30.6
02	52	20	38.5	25.3–53.0	250	50	20.0	15.2–25.5
03	166	35	21.1	15.2–28.1	823	184	22.4	19.6–25.4
04	120	26	21.7	14.7–30.1	665	137	20.6	17.6–23.9
05	92	22	23.9	15.6–33.9	601	70	11.7	9.2–14.5
06	355	97	27.3	22.8–32.3	1,481	242	16.3	14.5–18.3
07	93	14	15.1	8.5–24.0	474	46	9.7	7.2–12.7
08	24	1	4.2	0.1–21.1	122	23	18.9	12.3–26.9
09	6	2	33.3	4.3–77.7	47	12	25.5	13.9–40.4
10	0	–	–	–	2	0	0.0	0.0–0.8
11	21	5	23.8	8.2–47.2	80	19	23.8	15.0–34.6
12	74	12	16.2	8.7–26.6	466	85	18.2	14.8–22.1
13	124	32	25.8	18.4–34.4	478	83	17.4	14.1–21.1
14	211	48	22.8	17.3–29.0	994	198	19.9	17.5–22.5
15	209	39	18.7	13.6–24.6	1,223	202	16.5	14.5–18.7
16	–	–	–	–	–	–	–	–
17	93	12	12.9	6.9–21.5	626	87	13.9	11.3–16.9

a *Exact 95% confidence intervals. For the estimation by administrative region, only ticks collected from animals who did not travel out of their administrative region of residence within 14 days of tick collection were used (n = 1,710 cats and 8,517 dogs). Administrative regions are: 01: Bas-Saint-Laurent, 02: Saguenay-Lac-Saint-Jean, 03: Capitale-Nationale, 04: Mauricie, 05: Estrie, 06: Montréal, 07: Outaouais, 08: Abitibi-Témiscamingue, 09: Côte-Nord, 10: Nord-du-Québec, 11: Gaspésie-Îles-de-la-Madeleine, 12: Chaudière-Appalaches, 13: Laval, 14: Lanaudière, 15: Laurentides, 16: Montérégie, 17: Centre-du-Québec*.

The proportion of cats and dogs infested with *A. phagocytophilum*-infected ticks was much less than those exposed to *B. burgdorferi*-infected ticks. For example, 0.8% to 5.2% of infested cats and 1.1% to 2.9% of infested dogs were bitten by *A. phagocytophilum*-infected ticks (Table 2). Regional differences were also noted. As few as 0% of cats in Côte-Nord and Saguenay-Lac-Saint-Jean to as many as 7.1% of cats in Bas-Saint-Laurent were exposed to *A. phagocytophilum*-infected ticks, although most confidence intervals were overlapping due to limited sample size. A similar range in proportion of infested dogs was noted in different regions (Table 2). The monthly proportions of cats and dogs infested with *A. phagocytophilum*-infected ticks is presented in Supplementary Table 1.

**Table 2:** Estimated risk of exposure to blacklegged ticks infected with *Anaplasma phagocytophilum* in infested cats and dogs per year and administrative region in Quebec, Canada from 2010 to 2017.

**Year and administrative region**	**Infested cats**				**Infested dogs**			
	**Total number**	**Number exposed to infected ticks**	**Risk of exposure**		**Total number**	**Number exposed to infected ticks**	**Risk of exposure**	
			**%**	**CI (95%)^a^**			**%**	**CI (95%)^a^**
**Year**								
2010	122	1	0.8	0.0–4.5	657	19	2.9	1.8–4.5
2011	266	7	2.6	1.1–5.4	1,207	23	1.9	1.2–2.9
2012	133	2	1.5	0.2–5.3	919	16	1.7	1.0–2.8
2013	213	3	1.4	0.3–4.1	1,271	15	1.2	0.7–1.9
2014	181	4	2.2	0.6–5.6	1,422	15	1.1	0.6–1.7
2015	192	10	5.2	2.5–9.4	1,371	22	1.6	1.0–2.4
2016	229	12	5.2	2.7–9.0	1,630	34	2.1	1.5–2.9
2017	438	9	2.1	0.9–3.9	2,296	61	2.7	2.0–3.4
Global	1,774	48	2.7	2.0–3.6	10,773	205	1.9	1.7–2.2
**Administrative region**								
01	70	5	7.1	2.4–15.9	185	8	4.3	1.9–8.3
02	52	0	0.0	0.0–6.9	250	6	2.4	0.9–5.2
03	166	3	1.8	0.4–5.2	823	21	2.6	1.6–3.9
04	120	7	5.8	2.4–11.7	665	14	2.1	1.2–3.5
05	92	5	5.4	1.8–12.2	601	6	1.0	0.4–2.2
06	355	8	2.3	1.0–4.4	1,481	24	1.6	1.0–2.4
07	93	3	3.2	0.7–9.1	474	2	0.4	0.1–1.5
08	24	1	4.2	0.1–21.1	122	0	0.0	0.0–3.0
09	6	0	0.0	0.0–45.9	47	1	2.1	0.1–11.3
10	0	–	–	–	2	0	0.0	0.0–84.2
11	21	1	4.8	0.1–23.8	80	6	7.5	2.8–15.6
12	74	1	1.4	0.0–7.3	466	9	1.9	0.9–3.6
13	124	3	2.4	0.5–6.9	478	9	1.9	0.9–3.5
14	211	3	1.4	0.3–4.1	994	20	2.0	1.2–3.1
15	209	4	1.9	0.5–4.8	1,223	26	2.1	1.4–3.1
16	–	–	–	–	–	–	–	–
17	93	4	4.3	1.2–10.7	626	10	1.6	0.8–2.9

a *Exact 95% confidence intervals. For the estimation by administrative region, only ticks collected from animals who did not travel out of their administrative region of residence within 14 days of tick collection were used (n = 1,710 cats and 8,517 dogs). Administrative regions are: 01: Bas-Saint-Laurent, 02: Saguenay-Lac-Saint-Jean, 03: Capitale-Nationale, 04: Mauricie, 05: Estrie, 06: Montréal, 07: Outaouais, 08: Abitibi-Témiscamingue, 09: Côte-Nord, 10: Nord-du-Québec, 11: Gaspésie-Îles-de-la-Madeleine, 12: Chaudière-Appalaches, 13: Laval, 14: Lanaudière, 15: Laurentides, 16: Montérégie, 17: Centre-du-Québec*.

Ticks collected from a total of 1,239 cats and 7,797 dogs were tested for *B. miyamotoi*. The proportion of cats exposed to *B. miyamotoi*-infected ticks varied from 3.5% in 2014 to 0.2% in 2017, with the highest prevalence (8.0%) observed in 2015. A similar pattern was observed in dogs (Supplementary Table 2). Among companion animals that did not travel outside of their administrative region of residence within 14 days of tick collection, exposure to *B. miyamotoi*-infected ticks occurred in 6 and 11 administrative regions for cats and dogs, respectively (Supplementary Table 2).

Exposure of cats or dogs to ticks infected with *B. microti* was a very rare event. Only one infested cat and five infested dogs were bitten by ticks infected with this pathogen. The cat had traveled to the United States prior to tick collection while all but one of the dogs had travel histories, namely: one traveled in the Montérégie region, another to Ontario, and the other two to the United States (Supplementary Table 3).

Pets' risk of exposure to these four pathogens through ticks was also estimated for all resident cats and dogs of the surveillance program participative regions, no matter their travel history. For all three studied bacteria and the parasite, the absolute difference between the estimated regional risk of exposure among all resident animals and estimated regional risk of exposure among resident animals that did not travel varied between 0 and 2.5%. However, larger differences were observed between the risk in resident animals for a specific region vs. the risk in non-resident animals that traveled to this region within 14 days of tick collection (Supplementary Tables 4–7).

### Risk Mapping and Spatiotemporal Clusters of Exposure to Infected Ticks

**Figure 3:**
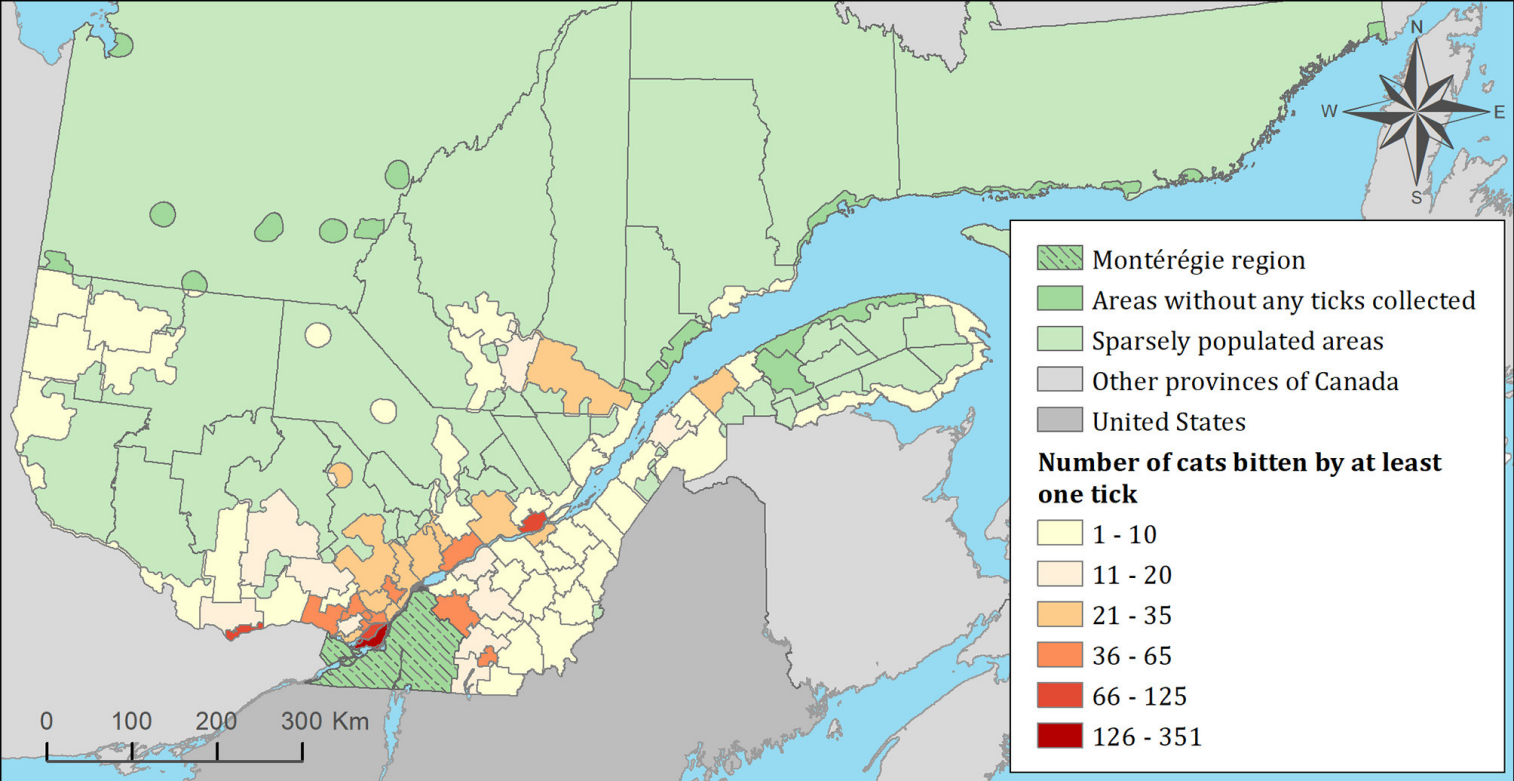
Number of cats infested with blacklegged ticks from 2010 to 2017 by regional county municipality considering the population ecumene (i.e., land with population density ≥0.4 persons per km^2^) and sparsely populated areas (i.e., unpopulated land or land with population density <0.4 persons per km^2^) of the province of Quebec, Canada. In total, 1,696 cats that did not travel out of their municipality of residence within 14 days of tick collection were included.

**Figure 4:**
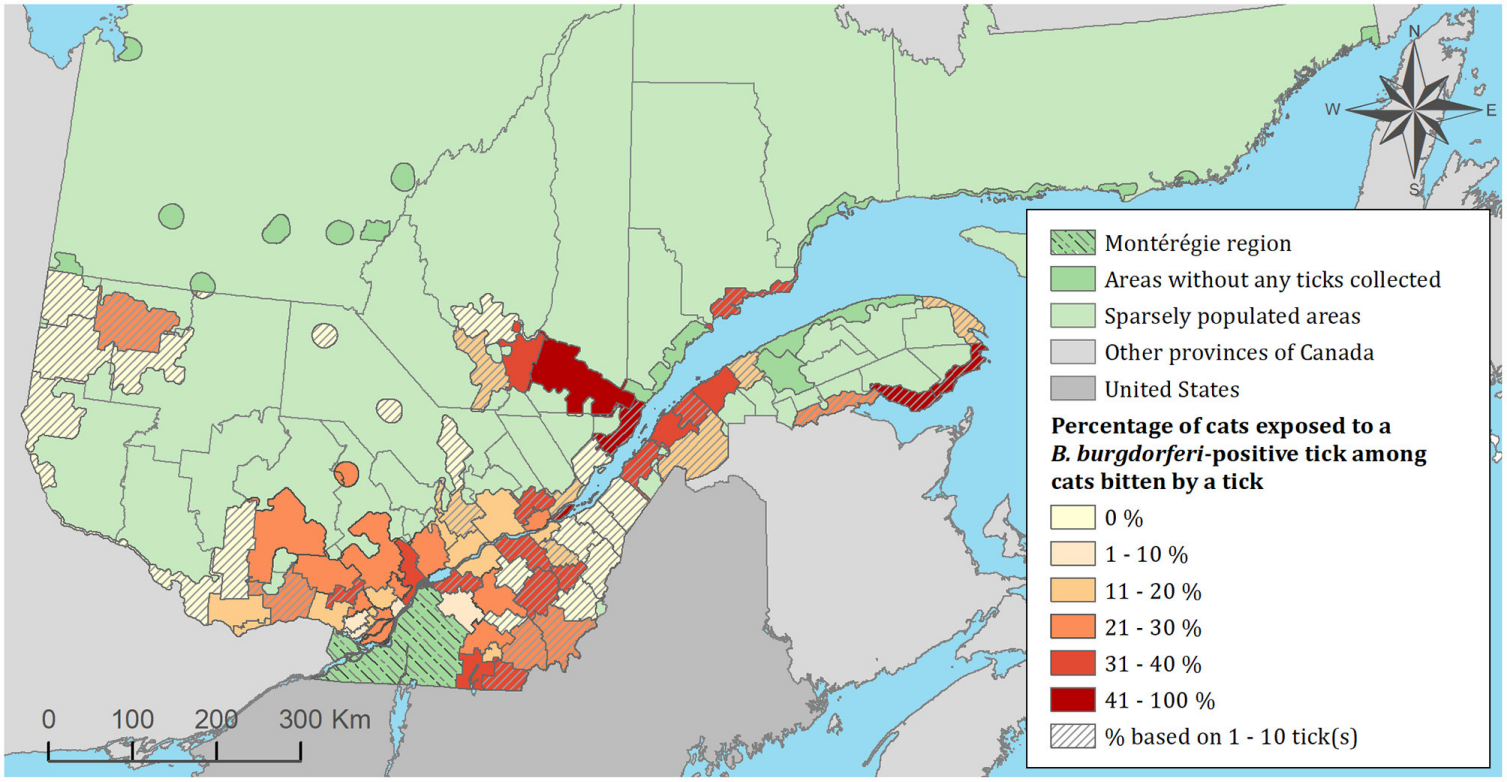
Percentage of cats exposed to *Borrelia burgdorferi* infected tick(s) among 1,696 cats infested with blacklegged ticks. Data covers from 2010 to 2017 by regional county municipality considering the population ecumene (i.e., land with population density ≥0.4 persons per km^2^) and sparsely populated areas (i.e., unpopulated land or land with population density <0.4 persons per km^2^) of the province of Quebec, Canada. Only cats that did not travel out of their municipality of residence within 14 days of tick collection were included.

**Figure 5:**
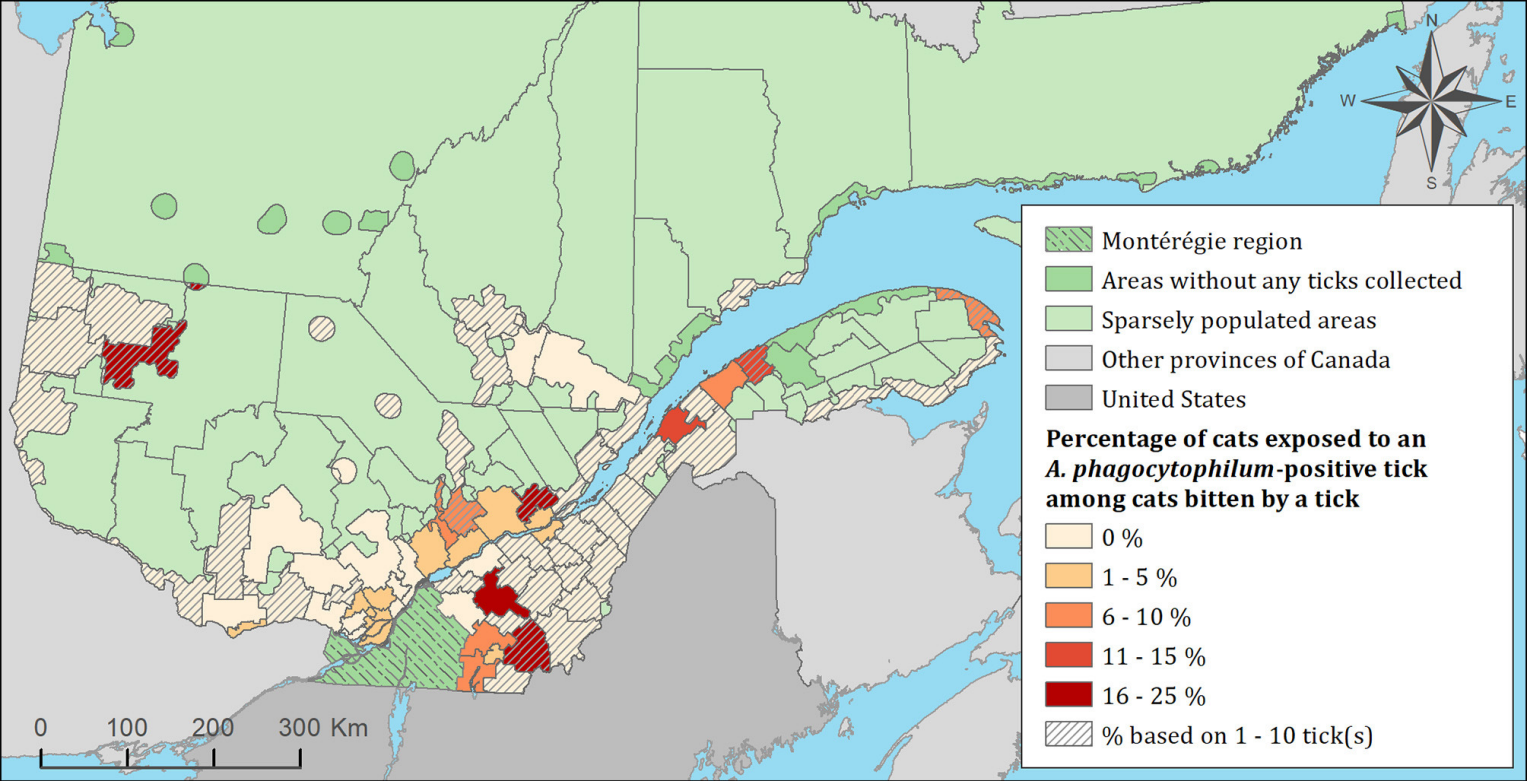
Percentage of cats exposed to *Anaplasma phagocytophilum* infected tick(s) among 1,696 cats infested with blacklegged ticks. Data covers from 2010 to 2017 by regional county municipality considering the population ecumene (i.e., land with population density ≥0.4 persons per km^2^) and sparsely populated areas (i.e., unpopulated land or land with population density <0.4 persons per km^2^) of the province of Quebec, Canada. Only cats that did not travel out of their municipality of residence within 14 days of tick collection were included.

**Figure 6:**
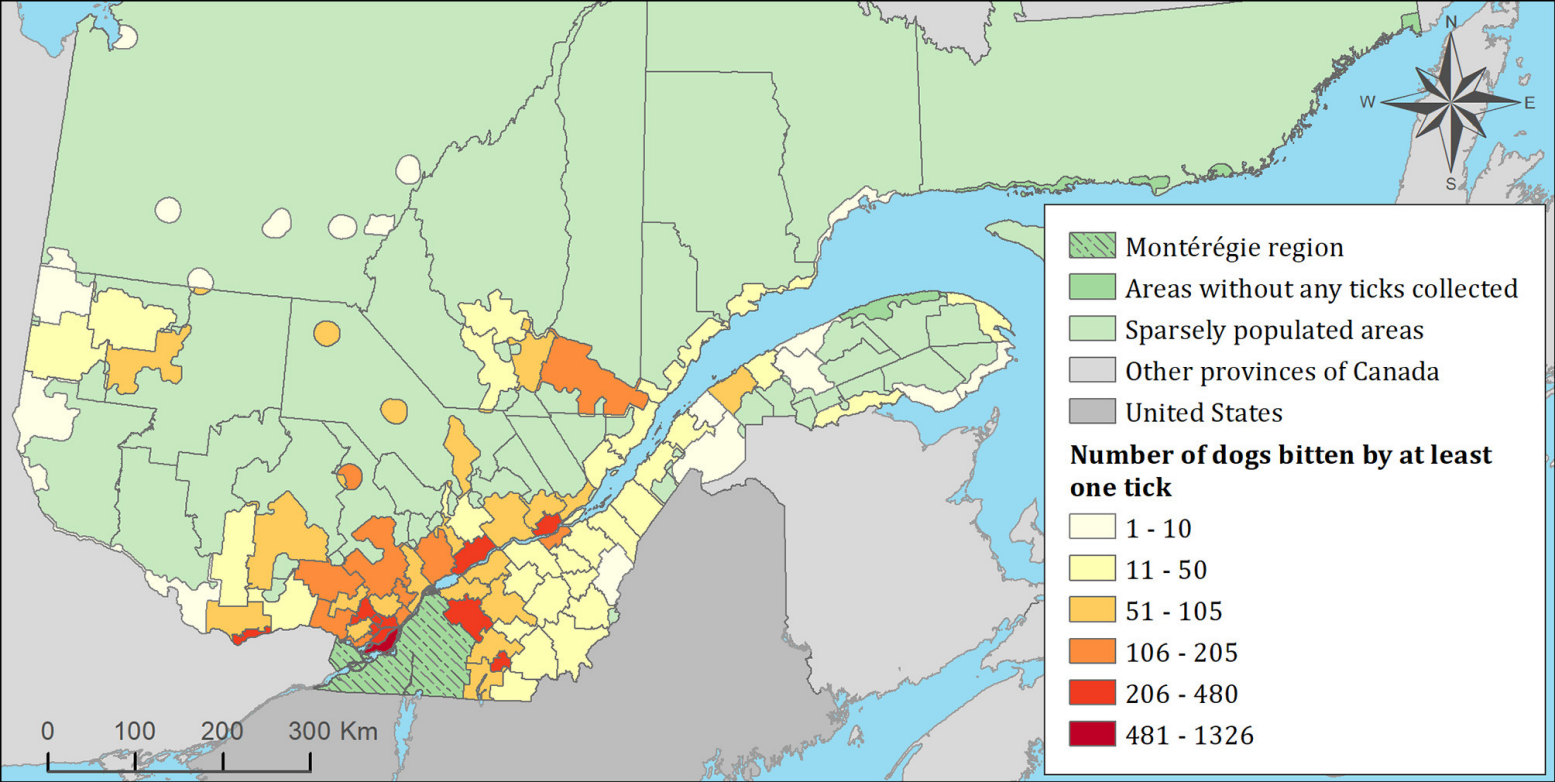
Number of dogs infested with blacklegged ticks from 2010 to 2017 by regional county municipality considering the population ecumene (i.e., land with population density ≥0.4 persons per km^2^) and sparsely populated areas (i.e., unpopulated land or land with population density <0.4 persons per km^2^) of the province of Quebec, Canada. In total, 7,644 dogs that did not travel out of their municipality of residence within 14 days of tick collection were included.

**Figure 7:**
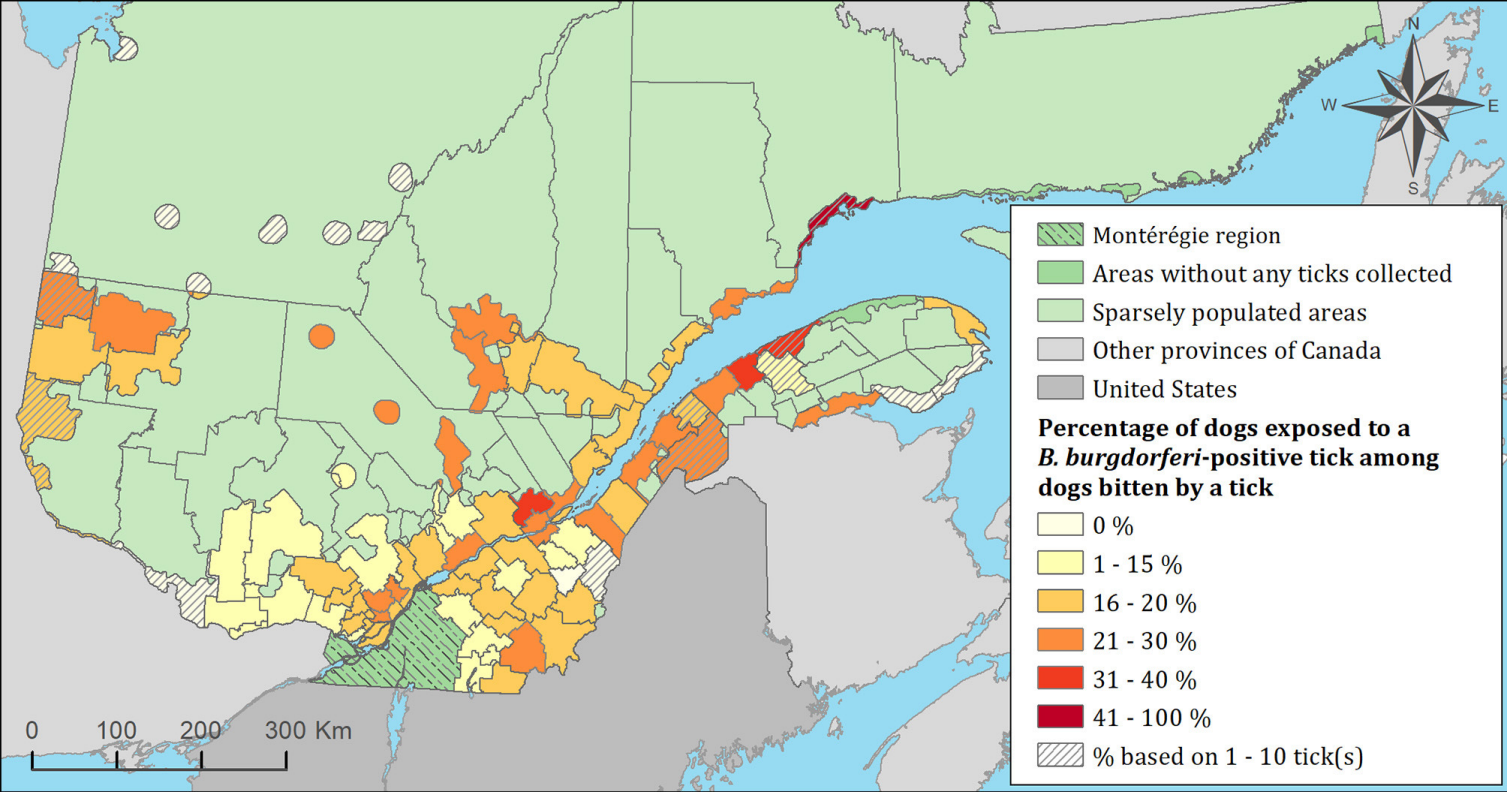
Percentage of dogs exposed to *Borrelia burgdorferi* infected tick(s) among 7,644 dogs infested with blacklegged ticks. Data cover from 2010 to 2017 by regional county municipality considering the population ecumene (i.e., land with population density ≥0.4 persons per km^2^) and sparsely populated areas (i.e., unpopulated land or land with population density <0.4 persons per km^2^) of the province of Quebec, Canada. Only dogs that did not travel out of their municipality of residence within 14 days of tick collection were included.

**Figure 8:**
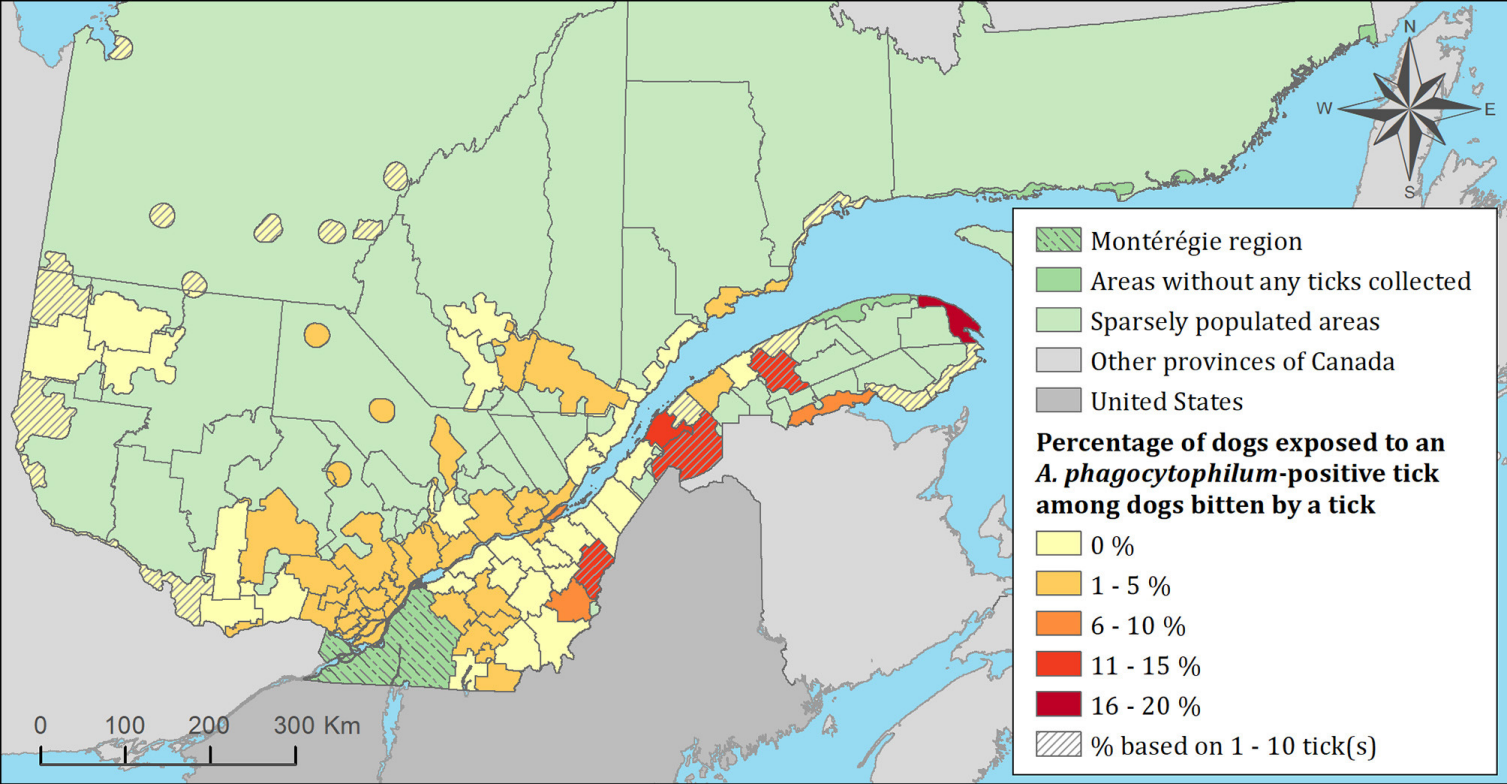
Percentage of dogs exposed to *Anaplasma phagocytophilum* infected tick(s) among 7,644 dogs infested with blacklegged ticks. Data cover from 2010 to 2017 by regional county municipality considering the population ecumene (i.e., land with population density ≥0.4 persons per km^2^) and sparsely populated areas (i.e., unpopulated land or land with population density <0.4 persons per km^2^) of the province of Quebec, Canada. Only dogs that did not travel out of their municipality of residence within 14 days of tick collection were included.

The spatial distribution of cats and dogs infested with blacklegged ticks infected with either *B. burgdorferi* or *A. phagocytophilum* are presented in Figures 3–8. In dogs, two spatiotemporal clusters were identified (Supplementary Figure 1 and Supplementary Table 8). The first cluster, which represents a higher risk area of exposure to positive ticks for *B. burgdorferi*, was located in the northern part of the province and was detected from 2011 to 2013. The other cluster of *B. burgdorferi* positivity was detected in 2015 in eastern Quebec, which only included 10 dogs. No statistically significant spatiotemporal high-risk clusters were identified for *A. phagocytophilum* or for cats.

### Factors Associated With Exposure of Hosts to Infected Ticks

For the analysis of exposure of cats and dogs to ticks infected with *B. burgdorferi*, the variables “condition of submitted ticks,” “quality of submitted ticks” and “month of tick collection” were not significant (*p*-value >0.05) and therefore excluded from the multivariable model. The final multivariable model demonstrated that the odds of exposure to tick(s) that were infected with *B. burgdorferi* was 2.3 (95% CI: 1.9–2.7) times higher for animals infested with multiple ticks (two or more ticks tested in pools) compared to animals infested with individual ticks, and 1.4 (95% CI: 1.2–1.5) times higher for cats than dogs (Table 3). Having traveled outside the province of Quebec within 14 days of tick collection was also identified as a risk factor for exposure to *B. burgdorferi*-infected ticks compared to cats and dogs that did not travel outside their municipality of residence, that traveled within their administrative region of residence, or outside their administrative region of residence but within the province (Table 3). Of note, of the 23 animals infested with *B. burgdorferi*-positive ticks collected from January to March, only six had traveled outside the province in the 14 days prior to tick collection, whereas 15 had not traveled outside the province, and travel histories were not available for two animals.

**Table 3:** Descriptive statistics of characteristics of submitted ticks, month of collection, host species and host travel history with *p*-value from univariable logistic regression and odd ratios (OR) with 95% confidence intervals (CI) and *p*-value from multivariable logistic regression modeling the exposure to blacklegged ticks infected with *Borrelia burgdorferi* in 12,547 infested animals in Quebec, Canada from 2010 to 2017.

**Characteristics**	**Number of infested animals**	**Exposure to infected ticks**		**Univariable analysis *p*-value^a^**	**Multivariable analysis**	
		**Number**	**%**		**OR (95% CI)**	***p*****-value^a^**
**Condition of submitted ticks^b^**				0.07		
At least one alive	384	89	23.2			
All dead	6,873	1,328	19.3			
**Quality of submitted ticks^b^**				0.16		
At least one intact	5,547	1,103	19.9			
All damaged	1,710	314	18.4			
**Maximum level of engorgement of submitted ticks^b^**				0.51		
Fully engorged	2,567	471	18.4			
Partially engorged	8,084	1,565	19.4			
Not engorged	375	70	18.7			
**Number of tick(s) pooled for PCR testing**				<0.001		
1	1,1920	2,153	18.1		ref.	
2 or more	627	226	36.0		2.3 (1.9–2.7)	<0.001
**Month of tick collection**				0.01		
January–March	100	23	23.0			
April–June	4,198	745	17.8			
July–September	545	89	16.3			
October–December	7,704	1,522	19.8			
**Host animal**				<0.001		
Cat	1,774	401	22.6		1.4 (1.2–1.5)	<0.001
Dog	10,773	1,978	18.4		ref.	
**Relative maximal travel distance for hosts within 14 days of tick collection^b^**				<0.001		
None/within municipality	9,340	1,710	18.3		0.5 (0.4–0.6)	<0.001
Out of municipality	887	158	17.8		0.5 (0.4–0.7)	<0.001
Out of administrative region	1,500	256	17.1		0.5 (0.4–0.6)	<0.001
Out of province	670	218	32.5		ref.	

a *Likelihood ratio test p-value. The final model includes the 12,397 animals with no missing values for all retained variables*.

b *Information was missing on the condition and quality of ticks from 5,290 animals, on level of engorgement for ticks from 1,521 animals, and on travel histories of 150 hosts*.

For the analysis of exposure of cats and dogs to ticks infected with *A. phagocytophilum*, the variables “host animal” and “condition of submitted ticks” were excluded from the multivariable model since they were not significant (*p*-value >0.05). The final multivariable model showed that the odds of exposure to ticks infected with *A. phagocytophilum* were 1.9 (95% CI: 1.2–2.9) times higher for animals infested with multiple ticks compared to animals exposed to individual ticks. Moreover, the odds of exposure for animals that traveled outside of the province of Quebec within 14 days of tick collection were 5 (95% CI: 2–14) times higher in comparison to animals that only traveled within their administrative region of residence, and 2.5 (95% CI: 1.4–5) times higher compared to animals that traveled to other administrative region(s) but within the province (Table 4).

**Table 4:** Descriptive statistics of characteristics of submitted ticks, month of collection, host species, and host travel history with *p*-value from univariable logistic regression and odd ratios (OR) with 95% confidence intervals (CI) and *p*-value from multivariable logistic regression modeling the exposure to blacklegged ticks infected with *Anaplasma phagocytophilum* in 12,547 infested animals in Quebec, Canada from 2010 to 2017.

**Characteristics**	**Number of infested animals**	**Exposure to infected ticks**		**Univariable analysis** ***p*****-value^a^**	**Multivariable model**	
		**Number**	**%**		**OR (95% CI)**	***p*****-value**
**Condition of submitted ticks^b^**				0.15		
At least one alive	384	13	3.4			
All dead	6,873	150	2.2			
**Quality of submitted ticks^b^**				0.77		
At least one intact	5,547	123	2.2			
All damaged	1,710	40	2.3			
**Maximum level of engorgement of submitted ticks^b^**				0.42		
Fully engorged	2,567	57	2.2			
Partially engorged	8,084	161	2.0			
Not engorged	375	11	2.9			
**Number of tick(s) pooled for PCR testing**				0.006		
1	11,920	230	1.9		ref.	
2 or more	627	23	3.7		1.9 (1.2–2.9)	0.007
**Month of tick collection**				0.25		
January–March	100	4	4.0			
April–June	4,198	73	1.7			
July–September	545	12	2.2			
October–December	7,704	164	2.1			
**Host animal**				0.03		
Cat	1,774	48	2.7			
Dog	10,773	205	1.9			
**Relative maximal travel distance for hosts within 14 days of tick collection^b^**				<0.001		
None/within municipality	9,340	205	2.2		0.8 (0.5–1.2)	0.23
Out of municipality	887	5	0.6		0.2 (0.1–0.5)	0.001
Out of administrative region	1,500	18	1.2		0.4 (0.2–0.7)	0.004
Out of province	670	22	3.3		ref.	

a *Likelihood ratio test p-value. The final model includes the 12,397 animals with no missing values for all retained variables*.

b *Information was missing on condition and quality of ticks from 5,290 animals, on level of engorgement of ticks from 1,521 animals, and on travel histories of 150 animals*.

For *B. miyamotoi*, only the month of tick collection was statistically significant in the univariable analysis. The odds of exposure of cats or dogs to ticks infected with *B. miyamotoi* were 12.6 (95% CI: 2.8–57.0) times higher for animals exposed to tick(s) from January to March compared to animals exposed to ticks from October to December (Supplementary Table 9).

For *B. microti*, only the number of ticks collected per animal and the travel history were significant in the univariable analysis. The odds of cats or dogs being exposed to ticks infected with *B. microti* was 18.6 (95% CI: 3.8–92.6) times higher for pets that were infested with multiple ticks compared to animals bitten by a single tick. Animals that traveled out of the province of Quebec within 14 days of tick collection had 66.9 (95% CI: 7.5–599.7) times the odds of exposure to *B. microti*-infected ticks in comparison to animals that did not travel (Supplementary Table 10).

### Coexposures of Hosts to Multiple Tick-Borne Pathogens

During this study, 2,581 (20.6%) of the 12,547 infested cats or dogs were bitten by ticks infected with at least one of the four pathogens of interest (Table 5). The various combinations of pathogens that hosts were exposed to are presented in Table 5. None of the cats or dogs were concurrently exposed to all four pathogens through the bites of one or multiple ticks.

**Table 5:** Description of exposure and coexposures of cats and dogs to blacklegged ticks infected with *Borrelia burgdorferi* (*Bb*), *Anaplasma phagocytophilum* (*Ap*), *Borrelia miyamotoi* (*Bmy*), and/or *Babesia microti* (*Bmc*) in Quebec, Canada from 2010 to 2017.

**Pathogens detected**	**Infested cats**		**Infested dogs**	
	**Number of hosts**	**Number exposed to infected ticks (%)**	**Number of hosts**	**Number exposed to infected ticks (%)**
*Bb*	1,774	401 (22.6%)	10,773	1,978 (18.4%)
*Ap*	1,774	48 (2.71%)	10,773	205 (1.90%)
*Bmy*	720	9 (1.25%)	3,963	49 (1.24%)
*Bmc*	1,239	1 (0.08%)	7,797	5 (0.06%)
*Bb* + *Ap*	1,774	17 (0.96%)	10,773	68 (0.63%)
*Bb* + *Bmy*	720	5 (0.69%)	3,963	19 (0.48%)
*Bb* + *Bmc*	1,239	1 (0.08%)	7,797	3 (0.04%)
*Ap* + *Bmy*	720	1 (0.14%)	3,963	4 (0.10%)
*Bb* + *Ap* + *Bmy*	720	0 (0.0%)	3,963	3 (0.08%)

In total, 1.7% of 10,168 animals bitten by *B. burgdorferi* PCR-negative ticks were exposed to *A. phagocytophilum*, compared to 3.6% of 2,379 animals bitten by *B. burgdorferi* PCR-positive ticks (*p* < 0.001). Similarly, the risk of exposure to *A. phagocytophilum* from a tick bite was 2.5% among the 4,625 animals bitten by *B. miyamotoi* PCR-negative ticks, compared to 8.6% among the 58 animals exposed to *B. miyamotoi* PCR-positive ticks (*p* = 0.02). Animals exposed to *B. burgdorferi* were also at higher risk of being concurrently exposed to *B. microti* (Supplementary Table 11).

### Veterinary Practices and Serological Results

The information regarding testing was only available for 124 cats in the study; only four veterinarians reported performing a serological test (one immunofluorescence assay, one SNAP 4DX and two undetermined tests) on the aforementioned cats. For dogs among which the information was available (i.e., tick testing positive for *B. burgdorferi* or *A. phagocytophilum* and voluntary completion and return of a questionnaire by the veterinarian), veterinarians reported performing a serological diagnostic test for 47% (423/908) of dogs bitten by ticks, with information on the diagnostic test performed available for 42% (378/908). Only the SNAP 4DX or SNAP 4DX Plus was used for 75% of the dogs, whereas 16% of dogs had a SNAP 4DX test performed in combination with a Lyme quantitative C_6_ antibody assay, and 9% of dogs only had the latter test. The date of completion of serological testing extended from the day of tick collection to over a year later, and the median was 74 days after tick collection. Other tests performed by veterinarians in combination with serological testing (or not) were urine analysis (*n* = 17), especially protein-creatinine ratio (*n* = 10), a complete blood count (*n* = 10), serum biochemistry (*n* = 7), and blood smears (*n* = 1).

Of the 310 dogs exposed to one or more ticks infected with *B. burgdorferi* and tested with the SNAP 4DX at least 4 weeks after tick collection, 111 (36%) had evidence of *B. burgdorferi* antibodies. Of the 82 dogs bitten by at least one *B. burgdorferi* positive tick on which a Lyme quantitative C_6_ antibody assay was performed at least 3 weeks after tick collection, 62 (76%) obtained a positive result, and 9 of 54 of these dogs (with available information) developed clinical signs consistent with LD according to their veterinarian. Both the SNAP 4Dx and Lyme Quant C_6_ were performed on 51 dogs; a positive result was obtained for both diagnostic tests for 41 of these dogs, both tests were negative for three dogs, while for the remaining seven dogs SNAP 4DX was positive and the Lyme Quant C6 was negative. Antibodies against *A. phagocytophilum* were detected in 34% (16/47) of dogs bitten by ticks infected with *A. phagocytophilum* and tested with a SNAP 4DX. Of these dogs, one developed clinical signs consistent with anaplasmosis according to their veterinarian. No information was available on the *B. burgdorferi* or *A. phagocytophilum* antibody status of these animals prior to tick collection.

The probability of detecting antibodies against *B. burgdorferi* in dogs bitten by *B. burgdorferi-*infected ticks was not associated with the maximum level of tick engorgement (Table 6). However, the odds of seropositivity to *B. burgdorferi* were 2.5 (95% CI: 1.1–5.8) times higher for dogs from which multiple ticks were collected compared to dogs from which only one tick was collected (Table 6).

**Table 6:** Descriptive statistics of characteristics of ticks collected and *p*-value from univariable logistic regression modeling the seropositivity to *Borrelia burgdorferi* in dogs infested with blacklegged ticks infected with *B. burgdorferi* in Quebec, Canada from 2010 to 2017.

**Characteristics**	**Number of dogs infested with ***B. burgdorferi*** PCR-positive ticks**	**Seropositive dogs^a^**		**Univariable analysis**	
		**Number**	**%**	**OR (95% CI)**	***p*****-value^b^**
**Maximum level of engorgement of submitted ticks*****^c^***					0.70
Fully engorged	75	30	40.0	1.1 (0.7–1.9)	
Partially engorged	232	89	38.4	ref.	
Not engorged	5	0	0.0		
**Number of ticks collected and pooled for PCR testing**					0.03
1	317	118	37.2	ref.	
2 or more	25	15	60.0	2.5 (1.1–5.8)	

a *B. burgdorferi serostatus of dogs based on a SNAP 4DX performed at least 4 weeks after tick collection and/or a Lyme quantitative C6 antibody assay was performed at least 3 weeks after collection of ticks*.

b *P-value are from likelihood ratio test*.

c *For the univariable analysis, the “Not engorged” and “Partially engorged” categories were merged to remove the categories without any observations to permit model convergence*.

Out of 673 dogs bitten by ticks only infected with *B. burgdorferi* and with available information on treatment, 77 (11%) were treated; 27 within 2 months of tick collection, 45 between 2 and 5 months after tick collection and three at least 5 months after tick collection (date of treatment was missing for two dogs). Among veterinarians who indicated the antibiotic treatment they administered (*n* = 9), doxycycline (*n* = 8) was the most common antibiotic used. Information on treatment was available for 124 dogs bitten by ticks only infected with *B. burgdorferi* and who had positive serology to this agent. Among these dogs, 48 (39%) were treated; 12 within 2 months of tick collection, 35 between 2 to 5 months of tick collection, and the other one at least 5 months after tick collection. Information on the presence of clinical signs compatible with LD and treatment was available for 113 dogs bitten by a tick only infected with *B. burgdorferi* and seropositive for this pathogen. Among those, 65% (11/17) of dogs that displayed clinical signs of LD and 31% (30/96) of dogs that were asymptomatic were treated. The majority of the clinical signs compatible with LD were observed between 2 and 5 months after tick collection. Out of 38 dogs bitten by ticks only infected with *A. phagocytophilum* and with available information on treatment, five (13%) were treated; four within 2 months of tick collection and the other one between 2 and 5 months after tick collection. Only one veterinarian indicated the antibiotic treatment they administered, marbofloxacin and amoxicillin. Information on treatment was available for five dogs bitten by ticks only infected with *A. phagocytophilum* and who were seropositive to this agent. Among these dogs, two (40%) were treated; one within 2 months of tick collection, and the other one between 2 and 5 months after tick collection. For both dogs, no clinical signs compatible with anaplasmosis were reported by the veterinarians before they administered the treatment. Among the 32 dogs bitten by ticks concurrently infected with *B. burgdorferi* and *A. phagocytophilum* with available information on treatment, six (19%) were treated; three within 2 months of tick collection and two between 2 and 5 months after tick collection. Half of the treated dogs had a positive serological test for *B. burgdorferi* and/or *A. phagocytophilum*, and none presented clinical signs compatible with LD or anaplasmosis based on the available data.

Information on vaccination against LD was available for 712 dogs bitten by *B. burgdorferi*-positive ticks; 2% (*n* = 14) were vaccinated for LD within a year before tick collection, 1% (*n* = 8) had been vaccinated more than 1 year prior to tick collection, 1% (*n* = 8) were vaccinated but no dates were given, and 96% (*n* = 682) were not vaccinated. Among non-vaccinated dogs, 4.3% (*n* = 29) were vaccinated for LD after tick collection.

## Discussion

Our findings represent a portrait of exposure to tick-borne pathogens in cats and dogs bitten by blacklegged ticks submitted to passive tick surveillance between 2010 and 2017 in the province of Quebec, Canada. The vast majority of ticks were collected from dogs, and most animals were infested with only one tick at the time of examination. By the time of removal, most ticks were partially or fully engorged, indicative of a recent blood meal (43). Most ticks were adult females, which is consistent with previous studies, and this is likely due in part to an increased ability to notice and remove adult females, which are much larger than nymphs (44, 45); differences in host preference between adult and immature stages of the blacklegged tick could also be involved as reported for other species (46). Interestingly, the bimodal seasonal pattern of infestations of cats and dogs was very similar to the pattern observed in humans from the same area and corresponds to the period of peak host-seeking for adult ticks in Quebec (45). Although we do not have a clear explanation for the lower amplitude of the spring seasonal peak in cats, it could be related to a hypothetical lower propensity for cats to spend time outdoors in risk areas in spring than dogs.

Interestingly, risk of exposure to *B. burgdorferi* in pets infested with blacklegged ticks in Quebec did not increase significantly between 2010 and 2017, nor did *B. burgdorferi* present a higher risk in southern areas which are more suitable for tick population establishment. Similarly, a temporal increase of the risk of exposure to blacklegged ticks infected with *A. phagocytophilum* and *B. miyamotoi* was not observed for infested cats and dogs. The absence of temporal increase of exposure to infected ticks is in marked contrast to earlier studies in which there was an increase in *B. burgdorferi* and *A. phagocytophilum* seropositivity reported in dogs between 2008 and 2015 in Quebec (30). However, a potential explanation is the exclusion of the Montérégie area from the study, which encompasses the large majority of municipalities considered at significant risk for LD during the study period in Quebec. Indeed, ticks collected in our study likely include adventitious ticks that have dropped off from migratory birds during seasonal patterns of spring migration (47). The overall risk of dogs being exposed to blacklegged ticks infected with *B. burgdorferi* was 18.4%, which is similar to the 15.4% infection rate reported in ticks collected from migratory birds in Canada (48), and is much lower than the >60% prevalence in ticks collected from sentinel surveillance sites from known LD-endemic areas in Quebec (49). Moreover, we do not have a clear explanation for the presence of the spatial and temporal cluster of *B. burgdorferi*-infected ticks observed in this study. However, considering that these clusters were either very limited in time and geographic space (i.e., one cluster was only present in 2015 and included 10 dogs) or located in northern areas, they are suggestive of a punctual increase in adventitious ticks, perhaps due to natural variation in migratory bird patterns and/or in the relative abundance of ticks infesting these hosts. Although infected adventitious ticks are not considered to constitute a risk for the establishment of an endemic transmission cycle of the pathogens of interest here (50), they do pose a health risk for the animals that they feed upon. Nevertheless, it should be noted that a high prevalence of pathogens in local ticks does not necessarily translate directly into a high regional risk of infection in pets, as this risk also depends on the likelihood of exposure to ticks. This could not be evaluated in our study as we did not have access to the regional pet population numbers of those actively involved in this tick surveillance program. However, our results are very likely representative of the regional risk of exposure to pathogens among animals that are bitten by blacklegged ticks. The very low risk of exposure to blacklegged ticks infected with *B. microti* during the study period suggests that this parasite did not constitute a significant risk in the province, and this is in agreement with a previous report (51).

The odds of exposure to blacklegged ticks infected with *B. burgdorferi, A. phagocytophilum, B. microti* and *B. miyamotoi* were always higher when ticks were pooled for testing and this is most likely due to an increased probability of detecting infected ticks in pooled samples. As an alternative hypothesis, animals with several ticks could be more likely to have picked them up from well-established tick populations with higher local infection rates. Interestingly, neither condition nor engorgement level had a significant effect on the prevalence of pathogens. Inconsistent findings have previously been reported on the impact of tick engorgement on the proportion of ticks infected with *B. burgdorferi* (43, 47, 52). Fewer damaged ticks were infected with *B. burgdorferi* but not the other pathogens and as a result, intact ticks should be prioritized for testing.

In our study, risk of exposure to blacklegged ticks infected with *B. burgdorferi* was significantly higher for infested dogs than cats. Ogden et al. observed a similar pattern and speculated that this may be due to anti-OspA present in vaccinated dogs clearing *B. burgdorferi* in a number of infected ticks (43, 53). That said, the data provided in our study does not allow us to draw conclusions regarding vaccine outcome. More studies are also warranted to draw definite conclusions on the effects of *B. burgdorferi* in cats.

Importantly, we observed that dogs which traveled outside of Quebec had a significantly higher risk of exposure to infected ticks (regardless of the pathogen) compared to dogs that did not travel. Tick populations have been well-established and extending their geographic range in Ontario and the United States for years (27, 44). It has been repeatedly demonstrated that *B. burgdorferi* infection prevalence increases in established tick populations, making areas with long-endemic tick population inherently of higher risk (43, 54). This information reinforces the importance of tick checks and the use of tick infestation preventative measures for animals traveling outside of Quebec. Within Quebec, we also explored the regional risk of exposure to infected ticks for resident animals that did not travel compared to the risk of exposure for non-resident animals that visited the same region, considering only travel history within the 14 days of tick collection. Interestingly, the risk was generally lower for non-resident animals, suggesting that in the context of a surveillance program, it might be important to consider the travel history of animals when presenting regional risk of exposure. This lower risk might be driven by a more frequent use of tick control products by dog owners used to traveling with their animals.

Given the diversity of pathogens with which blacklegged ticks can be infected, coinfections have been a theoretical concern for hosts exposed to multiple pathogens. For example, in humans, coinfections may result in a more serious illness and prolonged clinical signs (55). Likewise, infection with *A. phagocytophilum* has been reported to impair the immune system in infected mammals, possibly facilitating colonization with a second pathogen (56). Furthermore, these coinfections pose a diagnostic challenge for medical professionals (57). One meta-analysis reported rates of co-infection as high as 28% in some tick species (*Ixodes pacificus* and *Ixodes ricinus* were especially prone to coinfections), although coinfection and coexposure to pathogens was unpredictable across tick species and geographic regions (56). Our results demonstrate that coexposure to multiple pathogens occurred rather infrequently in Quebec. This observation seems logical given the overall low prevalence of pathogens other than *B. burgdorferi* in blacklegged ticks and this is consistent with results reported from other studies conducted in Canada (52). Despite this low prevalence, the probability of detecting a coexposure was higher than expected by chance for certain pathogen combinations, as also reported in other studies (52, 56, 58). The biological reason underlying this association is unclear. Of note, pets that were coexposed to *B. burgdorferi* and *B. microti* traveled outside the province of Quebec, mainly in the United-States, within 2 weeks of tick collection.

In our study, 47% of veterinarians that transmitted the information (on a voluntary basis) reported testing for antibodies in dogs bitten by ticks infected with *B. burgdorferi* and*/*or *A. phagocytophilum*. The vast majority were tested using the SNAP 4Dx (or SNAP 4Dx Plus) test, which is in accordance with the ACVIM 2018 guidelines as one of the validated serological tests for *B. burgdorferi*. Other tests performed included urine analysis and additional bloodwork; and these tests may be critically important to rule out proteinuria or hematological abnormalities in animals with antibodies against *B. burgdorferi* (1, 4). Information on serological testing was available for only a small number of cats (*n* = 124); however, four underwent serological tests; which is not surprising given the current lack of consensus on the impact of *B. burgdorferi* seropositivity in cats (1). Overall, 36% of dogs infested with *B. burgdorferi*-positive ticks were seropositive, suggesting that at most 36% seroconverted as we did not have access to serological status of dogs prior to tick collection. With respect to *A. phagocytophilum*, 34% of dogs infested with *A. phagocytophilum*-positive ticks were also seropositive for this pathogen. Cross-reactions among similar pathogens may play a role here. For example, positive results for *A. phagocytophilum* on serological testing may reflect exposure to *Anaplasma platys*, the widespread etiologic agent of cyclic thrombocytopenia, and consequently, results from serological testing need to be interpreted carefully (59). Data pertaining to clinical signs was limited given the voluntary nature of the questionnaire, so we could not use this information to account for possible cross-reactivity.

We observed that the odds of a dog testing seropositive for *B. burgdorferi* increased for dogs bitten by more than one tick, which could be due to exposure to an overall higher number of infected ticks. Another hypothesis is that dogs with multiple ticks are more likely to have had other attached ticks that went unnoticed in the past, increasing their chances of prior exposure to *B. burgdorferi*. Although owners may be more likely to notice ticks on their pets when more than one is present, their detection might take some time, especially in animals with thick fur. As *B. burgdorferi* transmission risk increases with increased tick attachment time, frequent tick checks and timely removal are critical (60). Management of LD is a complex topic that merits attention from general practitioners in the province. For example, recent studies involving family physicians in Quebec have revealed a number of misconceptions regarding the necessity of serological testing and antibiotic therapy in humans, as well as the value of tick testing in diagnosis and treatment of patients exposed to ticks (61, 62). These same issues are of concern among veterinarians in the province. Notably, the use of antibiotics in animals that are otherwise healthy but have antibodies against *B. burgdorferi* is still controversial. Recent ACVIM guidelines are clear on the value of regular *B. burgdorferi* screening for animals in endemic areas and testing *B. burgdorferi*-positive animals for proteinuria, but the treatment of healthy, seropositive dogs without clinical signs or proteinuria was a point of contention (1). The majority of panelists in the aforementioned paper do not recommend treatment in such cases. In our study, 11% of dogs infested with ticks infected only with *B. burgdorferi* received antibiotics, as well as 31% of dogs seropositive to *B. burgdorferi* that were bitten by ticks only infected with this pathogen but that did not present clinical signs of LD. This practice is counter to the current recommendations and likely has limited value since there is no evidence that antibiotics are effective in preventing future clinical signs. The ACVIM guidelines cite concerns over antibiotic overuse, lack of total parasite clearance, and possible reinfection as reasons not to treat a seropositive, clinically healthy animal. Furthermore, general practitioners are discouraged from prescribing antibiotics solely based on the outcomes of testing attached ticks (61). On the other hand, for animals clinically ill with LD or anaplasmosis, ACVIM guidelines recommend a 4-week course of antibiotics (1). In our study, 48% of dogs who developed clinical manifestations compatible with LD and/or anaplasmosis were treated, as were more specifically 65% of dogs bitten by ticks only infected with *B. burgdorferi* that also tested seropositive and that had clinical signs consistent with LD. Doxycycline was the most common antibiotic used, as recommended (1). Notably, some veterinarians reported the presence of clinical manifestations suggestive of LD <2 months after the tick collection, despite the experimental evidence of a long incubation period of 2 to 5 months in dogs (5). Although this might be reflective of veterinarian awareness regarding the large uncertainty surrounding the onset of LD or the knowledge gap on the onset of Lyme nephritis following infection in dogs, leading to a possibility that clinical signs can occur earlier, a misconception among veterinarians of LD evolution in dogs is also possible (5). Alternatively, veterinarians might have been aware of tick exposure in dogs earlier in the season, but this information was not reported to us or made available.

A LD vaccine has been available for dogs since 1990 and there are now numerous options available (1, 4). However, many of these vaccines have inconsistent efficacy and vaccination is also a highly debated topic. In our study, relatively few dogs were vaccinated, perhaps because of the uncertainty associated with their performance and utility. One systematic review and meta-analysis found that, although vaccines were significantly associated with lower odds of developing clinical LD in experimental studies, there are limitations in many studies including small sample size and potential bias (4, 63). Also, no information is available on the potential positive or negative impacts of vaccination on the risk of developing Lyme nephritis (5). Overall, half of 6 ACVIM panelists recommended routine use of LD vaccine in *B. burgdorferi-*endemic areas in North America (1, 4). In our study, information on vaccination against LD was unavailable for a large proportion of dogs; the data was therefore not substantial enough to draw significant conclusions with respect to vaccine efficacy. Interestingly, 4.3% of dogs were vaccinated *after* tick collection. This is in accordance with those ACVIM panelists who recommend vaccination against LD; they agreed that the vaccination of healthy, seropositive dogs may prove beneficial to decreasing the risk of reinfection (1). That being said, the use of tick infestation prevention products is to be prioritized (1, 4).

Some limitations should be taken into account in the interpretation of our results. First, underreporting of tick infestation is likely occurring, and this could be due in part to the voluntary nature of the participation by veterinary clinics and pet owners in our tick surveillance program. Representativeness may vary from one region of the province to another and across time depending on the proportion of participating clinics and owners, which may have been influenced by LD awareness campaigns; comparison between years and regions in the number of infested pets should therefore be interpreted cautiously. Considering that tick-borne diseases are evolving rapidly, the current portrait could also be different. Likewise, as no unique identification number for each pet was provided, we could not evaluate the likelihood of multiple submissions in time from the same animal, and thus each submission event had to be considered as independent. As most ticks were fully or partially engorged at time of collection, we cannot exclude that the detected pathogens originated from the host and not the tick. Veterinary management practices following the collection of a tick from cats or dogs could not be thoroughly described in this study because the questionnaire gathering this information was only distributed to clinics that submitted ticks that were found positive for *B. burgdorferi* or *A. phagocytophilum* by PCR. The information compiled in this study also does not thoroughly represent the management practices that veterinarians put in place when a tick is found positive, since the chronology of events could not be reliably determined. Indeed, we could not differentiate with confidence whether the serological testing or treatments were undertaken before or after the tick positive result was received by the clinic. Additionally, the completion of the questionnaire was done on a voluntary basis. This may have resulted in an overestimation of the proportion of veterinarians applying specific practices for LD management after tick removal, as these veterinarians may have been more inclined to fill out the questionnaire compared to others. Another limitation concerns the information from our study on clinical signs, since LD clinical signs are not specific, the ones reported in this study could not be definitively associated with LD.

In conclusion, our results support that from 2010 to 2017, cats and dogs throughout Quebec were at risk of exposure to *A. phagocytophilum* and *B. burgdorferi* through the bites of infected blacklegged ticks. Approximately 23% of cats and 18% of dogs were bitten by blacklegged ticks infected with *B. burgdorferi*, and a smaller percentage of hosts were bitten by ticks infected with *A. phagocytophilum*. As a result, veterinarians should be well aware of these risks and work to remain up-to-date on recommended case management strategies for animals that test positive for LD. Our results also highlight a potential misconception among some veterinarians regarding the need for treatment of healthy *B. burgdorferi*-seropositive dogs.

## Data Availability Statement

The data analyzed in this study is subject to the following licenses/restrictions: The dataset was provided to the research team by the Laboratoire de santé publique du Québec (LSPQ) under a data sharing agreement, with permission limited to the sharing of analysis outcomes. The dataset can be made available upon request to the LSPQ. Requests to access these datasets should be directed to karine.thivierge@inspq.qc.ca.

## Author Contributions

KT, CF-P, and JA: conceptualization and supervision. LD, SG, and JA: data curation. LD and JA: formal analysis. LD and VW: writing—original draft preparation. LRL and AD provided oversight on all of the tick testing conducted in this study. All authors provided input as part of the manuscript preparation and editing and approved the submitted version.

## Conflict of Interest

The authors declare that this study received funding from Intervet Canada Corp., operating in Canada as Merck Animal Health. The funder was not involved in the study design, collection, analysis, interpretation of data or the writing of this article.

## Abbreviations

LD: Lyme disease.
