# Supplementary material for: Exposure to Tick-Borne Pathogens in Cats and Dogs Infested With Ixodes scapularis in Quebec: An 8-Year Surveillance Study
Source: Front Vet Sci. 2021 Jul 15;8:696815. doi: 10.3389/fvets.2021.696815 (PMC8321249; doi:10.3389/fvets.2021.696815)
Supplement: Supplementary file 1 [file Data_Sheet_1.pdf]

Supplementary Material

1 Supplementary Tables

Supplementary Table 1. Estimated risk of exposure to blacklegged ticks infected with *Borrelia burgdorferi* (*Bb*) and *Anaplasma phagocytophilum* (*Ap*) in 1,774 infested cats and 10,773 infested dogs by month of collection in Quebec, Canada from 2010-2017.

	**Infested cats**			**Infested dogs**		
**Month of collection**	**Total number**	**Number exposed to infected ticks with *Bb* (%)**	**Number exposed to infected ticks with *Ap* (%)**	**Total number**	**Number exposed to infected ticks with *Bb* (%)**	**Number exposed to infected ticks with *Ap* (%)**
January	0	-	-	32	8 (25.0)	2 (6.3)
February	0	-	-	6	3 (50.0)	0 (0.0)
March	6	2 (33.3)	0 (0.0)	56	10 (17.9)	2 (3.6)
April	60	10 (16.7)	3 (5.0)	699	120 (17.2)	13 (1.9)
May	185	35 (18.9)	10 (5.4)	2056	371 (18.0)	30 (1.5)
June	100	25 (25.0)	0 (0.0)	1098	184 (16.8)	17 (1.6)
July	38	8 (21.1)	1 (2.6)	264	38 (14.4)	3 (1.1)
August	12	0 (0.0)	0 (0.0)	53	8 (15.1)	1 (1.9)
September	49	11 (22.5)	2 (4.1)	129	24 (18.6)	5 (3.9)
October	706	175 (24.8)	11 (1.6)	3288	641 (19.5)	65 (2.0)
November	558	121 (21.7)	18 (3.2)	2816	513 (18.2)	60 (2.1)
December	60	14 (23.3)	3 (5.0)	276	58 (21.0)	7 (2.5)

Supplementary Table 2. Estimated risk of exposure to blacklegged ticks infected with *Borrelia miyamotoi* in infested cats and dogs per year and administrative region in Quebec, Canada from 2014-2017.

Year and administrative region	Infested cats				Infested dogs			
	Total number	Number exposed to infected ticks	Risk of exposure		Total number	Number exposed to infected ticks	Risk of exposure	
			%	CI (95%)[1]			%	CI (95%)[1]
**Year**[2]								
2014	57	2	3.5	0.4-12.1	335	10	3.0	1.4-5.4
2015	50	4	8.0	2.2-19.2	291	21	7.2	4.5-10.8
2016	175	2	1.1	0.1-4.1	1041	11	1.1	0.5-1.9
2017	438	1	0.2	0.0-1.3	2296	7	0.3	0.1-0.6
Global	720	9	1.3	0.6-2.4	3963	49	1.2	1.0-1.6
**Administrative region**[3]								
01-Bas-Saint-Laurent	43	2	4.7	0.6-15.8	97	2	2.1	0.3-7.3
02-Saguenay-Lac-Saint-Jean	29	0	0.0	0.0-11.9	139	0	0.0	0.0-2.6
03-Capitale-Nationale	78	0	0.0	0.0-4.6	356	3	0.8	0.2-2.4
04-Mauricie	36	1	2.8	0.1-14.5	248	2	0.8	0.1-2.9
05-Estrie	44	1	2.3	0.1-12.0	229	6	2.6	1.0-5.6
06-Montréal	115	1	0.9	0.0-4.8	439	8	1.8	0.8-3.6
07-Outaouais	26	0	0.0	0.0-13.2	143	2	1.4	0.2-5.0
08-Abitibi-Témiscamingue	12	0	0.0	0.0-26.5	81	0	0.0	0.0-4.5
09-Côte-Nord	2	0	0.0	0.0-84.2	20	1	5.0	0.1-24.9
10-Nord-du-Québec	0	--	--	--	2	0	0.0	0.0-84.2
11-Gaspésie-Îles-de-la-Madeleine	14	0	0.0	0.0-23.2	62	0	0.0	0.0-5.8
12-Chaudière-Appalaches	44	0	0.0	0.0-8.0	200	3	1.5	0.3-4.3
13-Laval	37	0	0.0	0.0-9.5	179	2	1.1	0.1-4.0
14-Lanaudière	87	1	1.2	0.0-6.2	411	7	1.7	0.7-3.5
15-Laurentides	93	3	3.2	0.7-9.1	442	4	0.9	0.3-2.3
16-Montérégie	--	--	--	--	--	--	--	--
17-Centre-du-Québec	32	0	0.0	0.0-10.9	192	0	0.0	0.0-1.9

[1] Exact 95% confidence intervals. [2] PCR testing for *B. miyamotoi* started in 2014. [3] For the estimation by administrative region, only ticks collected from animals that did not travel out of their administrative region of residence within 14 days of tick collection and that were tested for *B. miyamotoi* were used (n=692 cats and 3,240 dogs).

Supplementary Table 3. Estimated risk of exposure to blacklegged ticks infected with *Babesia microti* in infested cats and dogs per year and administrative region in Quebec, Canada from 2013-2017.

Year and administrative region	Infested cats				Infested dogs			
	Total number	Number exposed to infected ticks	Risk of exposure		Total number	Number exposed to infected ticks	Risk of exposure	
			%	CI (95%)[1]			%	CI (95%)[1]
**Year[2]**								
2013	199	0	0.0	0.0-1.8	1080	1	0.1	0.0-0.5
2014	181	0	0.0	0.0-2.0	1420	1	0.1	0.0-0.4
2015	192	0	0.0	0.0-1.9	1371	1	0.1	0.0-0.4
2016	229	0	0.0	0.0-1.6	1630	1	0.1	0.0-0.3
2017	438	1	0.2	0.0-1.3	2296	1	0.0	0.0-0.2
Global	1239	1	0.1	0.0-0.5	7797	5	0.1	0.0-0.2
**Administrative region[3]**								
01-Bas-Saint-Laurent	64	0	0.0	0.0-5.6	152	0	0.0	0.0-2.4
02-Saguenay-Lac-Saint-Jean	41	0	0.0	0.0-8.6	220	0	0.0	0.0-1.7
03-Capitale-Nationale	111	0	0.0	0.0-3.3	607	0	0.0	0.0-0.6
04-Mauricie	81	0	0.0	0.0-4.5	462	1	0.2	0.0-1.2
05-Estrie	68	0	0.0	0.0-5.3	478	0	0.0	0.0-0.8
06-Montréal	226	0	0.0	0.0-1.6	1018	0	0.0	0.0-0.4
07-Outaouais	59	0	0.0	0.0-6.1	313	0	0.0	0.0-1.2
08-Abitibi-Témiscamingue	20	0	0.0	0.0-16.8	109	0	0.0	0.0-3.3
09-Côte-Nord	4	0	0.0	0.0-60.2	37	0	0.0	0.0-9.5
10-Nord-du-Québec	0	--	--	--	2	0	0.0	0.0-84.2
11-Gaspésie-Îles-de-la-Madeleine	18	0	0.0	0.0-18.5	76	0	0.0	0.0-4.7
12-Chaudière-Appalaches	58	0	0.0	0.0-6.2	329	0	0.0	0.0-1.1
13-Laval	84	0	0.0	0.0-4.3	363	0	0.0	0.0-1.0
14-Lanaudière	150	0	0.0	0.0-2.4	754	0	0.0	0.0-0.5
15-Laurentides	145	0	0.0	0.0-2.5	878	0	0.0	0.0-0.4
16-Montérégie	--	--	--	--	--	--	--	--
17-Centre-du-Québec	56	0	0.0	0.0-6.4	436	0	0.0	0.0-0.8

[1]Exact 95% confidence intervals. [2] PCR testing for *B. microti* started in 2013. [3] For the estimation by administrative region, only ticks collected from animals that did not travel out of their administrative region of residence within 14 days of tick collection and that were tested for *B. microti* were used (n=1,185 cats and 6,234 dogs).

Supplementary Table 4. Estimated risk of exposure to blacklegged ticks infected with *Borrelia burgdorferi* in infested cats and dogs by host administrative region of residence and according to the host history of travel in Quebec, Canada from 2010-2017.

Animal species and administrative region	All resident[1]			Resident (no travel)[2]			Non-resident (travelers)[3]		
	Number exposed to infected ticks/total	Risk of exposure %	Risk of exposure CI (95%)[4]	Number exposed to infected ticks/total	Risk of exposure %	Risk of exposure CI (95%)[4]	Number exposed to infected ticks/total	Risk of exposure %	Risk of exposure CI (95%)[4]
**Cat**									
01-Bas-Saint-Laurent	21/71	29.6	19.3-41.6	21/70	30.0	19.6-42.1	0/3	0.0	0.0-70.8
02-Saguenay-Lac-Saint-Jean	20/52	38.5	25.3-53.0	20/52	38.5	25.3-53.0	0	--	--
03-Capitale-Nationale	37/176	21.0	15.3-27.8	35/166	21.1	15.2-28.1	0	--	--
04-Mauricie	26/121	21.5	14.5-29.9	26/120	21.7	14.7-30.1	0/1	0.0	0.0-97.5
05-Estrie	22/95	23.2	15.1-32.9	22/92	23.9	15.6-33.9	0	--	--
06-Montréal	101/381	26.5	22.1-31.2	97/355	27.3	22.8-32.3	1/2	50.0	1.3-98.7
07-Outaouais	15/95	15.8	9.1-24.7	14/93	15.1	8.5-24.0	0/2	0.0	0.0-84.2
08-Abitibi-Témiscamingue	1 /25	4.0	0.1-20.4	1/24	4.2	0.1-21.1	0	--	--
09-Côte-Nord	2/6	33.3	4.3-77.7	2/6	33.3	4.3-77.7	0	--	--
10-Nord-du-Québec	0	--	--	0	--	--	0	--	--
11-Gaspésie-Îles-de-la-Madeleine	5/21	23.8	8.2-47.2	5/21	23.8	8.2-47.2	0	--	--
12-Chaudière-Appalaches	12/76	15.8	8.4-26.0	12/74	16.2	8.7-26.6	2/3	66.7	9.4-99.2
13-Laval	32/129	24.8	17.6-33.2	32/124	25.8	18.4-34.4	0	--	--
14-Lanaudière	51/215	23.7	18.2-30.0	48/211	22.8	17.3-29.0	1/4	25.0	0.6-80.6
15-Laurentides	43/217	19.8	14.7-25.8	39/209	18.7	13.6-24.6	2/9	22.2	2.8-60.0
16-Montérégie	--	--	--	--	--	--	1/14	7.1	0.2-33.9
17-Centre-du-Québec	13/94	13.8	7.6-22.5	12/93	12.9	6.9-21.5	0	--	--
**Dog**									
01-Bas-Saint-Laurent	51/211	24.2	18.6-30.5	44/185	23.8	17.8-30.6	4/30	13.3	3.8-30.7
02-Saguenay-Lac-Saint-Jean	54/271	19.9	15.3-25.2	50/250	20.0	15.2-25.5	0/19	0.0	0.0-17.7
03-Capitale-Nationale	218/1003	21.7	19.2-24.4	184/823	22.4	19.6-25.4	9/46	19.6	9.4-33.9
04-Mauricie	164/760	21.6	18.7-24.7	137/665	20.6	17.6-23.9	7/68	10.3	4.2-20.1
05-Estrie	86/664	13.0	10.5-15.8	70/601	11.7	9.2-14.5	13/97	13.4	7.3-21.8

Animal species and administrative region	All resident[1]			Resident (no travel)[2]			Non-resident (travelers)[3]		
	Number exposed to infected ticks/total	Risk of exposure		Number exposed to infected ticks/total	Risk of exposure		Number exposed to infected ticks/total	Risk of exposure	
		%	CI (95%)[4]		%	CI (95%)[4]		%	CI (95%)[4]
06-Montréal	447/2480	18.0	16.5-19.6	242/1481	16.3	14.5-18.3	9/64	14.1	6.6-25.0
07-Outaouais	53/542	9.8	7.4-12.6	46/474	9.7	7.2-12.7	4/35	11.4	3.2-26.7
08-Abitibi-Témiscamingue	27/139	19.4	13.2-27.0	23/122	18.9	12.3-26.9	1/4	25.0	0.6-80.6
09-Côte-Nord	14/50	28.0	16.2-42.5	12/47	25.5	13.9-40.4	2/4	50.0	6.8-93.2
10-Nord-du-Québec	2/10	20.0	2.5-55.6	0/2	0.0	0.0-0.8	0/3	0.0	0.0-70.8
11-Gaspésie-Îles-de-la-Madeleine	19/85	22.4	14.0-32.7	19/80	23.8	15.0-34.6	0/4	0.0	0.0-60.2
12-Chaudière-Appalaches	97/533	18.2	15.0-21.7	85/466	18.2	14.8-22.1	6/53	11.3	4.3-23.0
13-Laval	125/632	19.8	16.7-23.1	83/478	17.4	14.1-21.1	5/33	15.2	5.1-31.9
14-Lanaudière	251/1209	20.8	18.5-23.2	198/994	19.9	17.5-22.5	21/126	16.7	10.6-24.3
15-Laurentides	274/1507	18.2	16.3-20.2	202/1223	16.5	14.5-18.7	24/191	12.6	8.2-18.1
16-Montérégie	--	--	--	--	--	--	98/459	21.4	17.7-25.4
17-Centre-du-Québec	96/677	14.2	11.6-17.0	87/626	13.9	11.3-16.9	7/49	14.3	5.9-27.2

[1] All resident cats (n=1,774) and dogs (n=10,773) of the administrative region, whatever their travel history.

[2] Only the resident cats (n=1,710) and dogs (n=8,517) that did not travel out of their administrative region of residence within 14 days of tick collection.

[3] Cats and dogs not resident of the administrative region but that traveled in the region within 14 days of tick collection. Only the cats (n=38) and dogs (n=1,285) that traveled outside their administrative region of residence, but only to a single other region within 14 days of tick collection (excludes cats and dogs that traveled to two or more other administrative regions).

[4] Exact 95% confidence intervals.

Supplementary Table 5. Estimated risk of exposure to blacklegged ticks infected with *Anaplasma phagocytophilum* in infested cats and dogs by host administrative region of residence and according to the host travel history in Quebec, Canada from 2010-2017.

Animal species and administrative region	All resident[1]			Resident (no travel)[2]			Non-resident (travelers)[3]		
	Number exposed to infected ticks/total	Risk of exposure		Number exposed to infected ticks/total	Risk of exposure		Number exposed to infected ticks/total	Risk of exposure	
		%	CI (95%)[4]		%	CI (95%)[4]		%	CI (95%)[4]
**Cat**									
01-Bas-Saint-Laurent	5/71	7.0	2.3-15.7	5/70	7.1	2.4-15.9	0/3	0.0	0.0-70.8
02-Saguenay-Lac-Saint-Jean	0/52	0.0	0.0-6.9	0/52	0.0	0.0-6.9	0	--	--
03-Capitale-Nationale	3/176	1.7	0.4-4.9	3/166	1.8	0.4-5.2	0	--	--
04-Mauricie	7/121	5.8	2.4-11.6	7/120	5.8	2.4-11.7	0/1	0.0	0.0-97.5
05-Estrie	5/95	5.3	1.7-11.9	5/92	5.4	1.8-12.2	0	--	
06-Montréal	8/381	2.1	0.9-4.1	8/355	2.3	1.0-4.4	0/2	0.0	0.0-84.2
07-Outaouais	3/95	3.2	0.7-9.0	3/93	3.2	0.7-9.1	0/2	0.0	0.0-84.2
08-Abitibi-Témiscamingue	1/25	4.0	0.1-20.4	1/24	4.2	0.1-21.1	0	--	--
09-Côte-Nord	0/6	0.0	0.0-45.9	0/6	0.0	0.0-45.9	0	--	--
10-Nord-du-Québec	0	--	--	0	--	--	0	--	--
11-Gaspésie-Îles-de-la-Madeleine	1/21	4.8	0.1-23.8	1/21	4.8	0.1-23.8	0	--	--
12-Chaudière-Appalaches	1/76	1.3	0.0-7.1	1/74	1.4	0.0-7.3	0/3	0.0	0.0-70.8
13-Laval	3/129	2.3	0.5-6.7	3/124	2.4	0.5-6.9	0	--	--
14-Lanaudière	3/215	1.4	0.3-4.0	3/211	1.4	0.3-4.1	0/4	0.0	0.0-60.2
15-Laurentides	4/217	1.8	0.5-4.7	4/209	1.9	0.5-4.8	0/9	0.0	0.0-33.6
16-Montérégie	--	--	--	--	--	--	0/14	0.0	0.0-23.2
17-Centre-du-Québec	4/94	4.3	1.2-10.5	4/93	4.3	1.2-10.7	0	--	--
**Dog**									
01-Bas-Saint-Laurent	10/211	4.7	2.3-8.5	8/185	4.3	1.9-8.3	0/30	0.0	0.0-11.6
02-Saguenay-Lac-Saint-Jean	8/271	3.0	1.3-5.7	6/250	2.4	0.9-5.2	0/19	0.0	0.0-17.7
03-Capitale-Nationale	23/1003	2.3	1.5-3.4	21/823	2.6	1.6-3.9	1/46	2.2	0.1-11.5
04-Mauricie	16/760	2.1	1.2-3.4	14/665	2.1	1.2-3.5	0/68	0.0	0.0-5.3
05-Estrie	8/664	1.2	0.5-2.4	6/601	1.0	0.4-2.2	0/97	0.0	0.0-3.7
06-Montréal	39/2480	1.6	1.1-2.1	24/1481	1.6	1.0-2.4	1/64	1.6	0.0-8.4

Animal species and administrative region	All resident[1]			Resident (no travel)[2]			Non-resident (travelers)[3]		
	Number exposed to infected ticks/total	Risk of exposure		Number exposed to infected ticks/total	Risk of exposure		Number exposed to infected ticks/total	Risk of exposure	
		%	CI (95%)[4]		%	CI (95%)[4]		%	CI (95%)[4]
07-Outaouais	2/542	0.4	0.0-1.3	2/474	0.4	0.1-1.5	0/35	0.0	0.0-10.0
08-Abitibi-Témiscamingue	1/139	0.7	0.0-3.9	0/122	0.0	0.0-3.0	0/4	0.0	0.0-60.2
09-Côte-Nord	1/50	2.0	0.0-10.7	1/47	2.1	0.1-11.3	1/4	25.0	0.6-80.6
10-Nord-du-Québec	0/10	0.0	0.0-30.9	0/2	0.0	0.0-84.2	0/3	0.0	0.0-70.8
11-Gaspésie-Îles-de-la-Madeleine	6/85	7.1	2.6-14.7	6/80	7.5	2.8-15.6	0/4	0.0	0.0-60.2
12-Chaudière-Appalaches	10/533	1.9	0.9-3.4	9/466	1.9	0.9-3.6	0/53	0.0	0.0-6.7
13-Laval	12/632	1.9	1.0-3.3	9/478	1.9	0.9-3.5	1/33	3.0	0.1-15.8
14-Lanaudière	26/1209	2.2	1.4-3.1	20/994	2.0	1.2-3.1	4/126	3.2	0.9-7.9
15-Laurentides	30/1507	2.0	1.4-2.8	26/1223	2.1	1.4-3.1	6/191	3.1	1.2-6.7
16-Montérégie	--	--	--	--	--	--	2/459	0.4	0.1-1.6
17-Centre-du-Québec	13/677	1.9	1.0-3.3	10/626	1.6	0.8-2.9	0/49	0.0	0.0-7.3

[1] All resident cats (n=1,774) and dogs (n=10,773) of the administrative region, whatever their travel history.

[2] Only the resident cats (n=1,710) and dogs (n=8,517) that did not travel out of their administrative region of residence within 14 days of tick collection.

[3] Cats and dogs not resident of the administrative region but that traveled in the region within 14 days of tick collection. Only the cats (n=38) and dogs (n=1,285) that traveled outside their administrative region of residence, but only to a single other region within 14 days of tick collection (excludes cats and dogs that traveled to two or more other administrative regions).

[4] Exact 95% confidence intervals.

Supplementary Table 6. Estimated risk of exposure to blacklegged ticks infected with *Borrelia miyamotoi* in infested cats and dogs by host administrative region of residence and according to the host travel history in Quebec, Canada from 2014-2017.

Animal species and administrative region	All resident[1]			Resident (no travel)[2]			Non-resident (travelers)[3]		
	Number exposed to infected ticks/total	Risk of exposure		Number exposed to infected ticks/total	Risk of exposure		Number exposed to infected ticks/total	Risk of exposure	
		%	CI (95%)[4]		%	CI (95%)[4]		%	CI (95%)[4]
**Cat**									
01-Bas-Saint-Laurent	2/44	4.6	0.6-15.5	2/43	4.7	0.6-15.8	0	--	--
02-Saguenay-Lac-Saint-Jean	0/29	0.0	0.0-11.9	0/29	0.0	0.0-11.9	0	--	--
03-Capitale-Nationale	0/84	0.0	0.0-4.3	0/78	0.0	0.0-4.6	0	--	--
04-Mauricie	1/37	2.7	0.1-14.2	1/36	2.8	0.0-14.5	0/1	0.0	0.0-97.5
05-Estrie	1/46	2.2	0.1-11.5	1/44	2.3	0.1-12.0	0	--	--
06-Montréal	1/120	0.8	0.0-4.6	1/115	0.9	0.0-4.8	0/1	0.0	0.0-97.5
07-Outaouais	0/27	0.0	0.0-12.8	0/26	0.0	0.0-13.2	0	--	--
08-Abitibi-Témiscamingue	0/13	0.0	0.0-24.7	0/12	0.0	0.0-26.5	0	--	--
09-Côte-Nord	0/2	0.0	0.0-84.2	0/2	0.0	0.0-84.2	0	--	--
10-Nord-du-Québec	0	--	--	0	--	--	0	--	--
11-Gaspésie-Îles-de-la-Madeleine	0/14	0.0	0.0-23.2	0/14	0.0	0.0-23.2	0	--	--
12-Chaudière-Appalaches	0/45	0.0	0.0-7.9	0/44	0.0	0.0-8.0	0/3	0.0	0.0-70.8
13-Laval	0/39	0.0	0.0-9.0	0/37	0.0	0.0-9.5	0	--	--
14-Lanaudière	1/89	1.1	0.0-6.1	1/87	1.2	0.0-6.2	0/2	0.0	0.0-84.2
15-Laurentides	3/98	3.1	0.6-8.7	3/93	3.2	0.7-9.1	0/2	0.0	0.0-84.2
16-Montérégie	--	--	--	--	--	--	0/6	0.0	0.0-45.9
17-Centre-du-Québec	0/33	0.0	0.0-10.6	0/32	0.0	0.0-10.9	0	--	--
**Dog**									
01-Bas-Saint-Laurent	2/110	1.8	0.2-6.4	2/97	2.1	0.3-7.3	0/16	0.0	0.0-20.6
02-Saguenay-Lac-Saint-Jean	0/150	0.0	0.0-2.4	0/139	0.0	0.0-2.6	0/10	0.0	0.0-30.9
03-Capitale-Nationale	4/426	0.9	0.3-2.4	3/356	0.8	0.2-2.4	0/14	0.0	0.0-23.2
04-Mauricie	2/281	0.7	0.1-2.6	2/248	0.8	0.1-2.9	1/21	4.8	0.1-23.8
05-Estrie	6/258	2.3	0.9-5.0	6/229	2.6	1.0-5.6	1/27	3.7	0.1-19.0
06-Montréal	10/678	1.5	0.7-2.7	8/439	1.8	0.8-3.6	0/27	0.0	0.0-12.8

Animal species and administrative region	All resident[1]			Resident (no travel)[2]			Non-resident (travelers)[3]		
	Number exposed to infected ticks/total	Risk of exposure		Number exposed to infected ticks/total	Risk of exposure		Number exposed to infected ticks/total	Risk of exposure	
		%	CI (95%)[4]		%	CI (95%)[4]		%	CI (95%)[4]
07-Outaouais	3/158	1.9	0.4-5.5	2/143	1.4	0.2-5.0	1/10	10.0	0.3-44.5
08-Abitibi-Témiscamingue	1/89	1.1	0.0-6.1	0/81	0.0	0.0-4.5	0/3	0.0	0.0-70.8
09-Côte-Nord	1/22	4.6	0.1-22.8	1/20	5.0	0.1-24.9	0/3	0.0	0.0-70.8
10-Nord-du-Québec	0/4	0.0	0.0-60.2	0/2	0.0	0.0-84.2	0/3	0.0	0.0-70.8
11-Gaspésie-Îles-de-la-Madeleine	0/67	0.0	0.0-5.4	0/62	0.0	0.0-5.8	0/1	0.0	0.0-97.5
12-Chaudière-Appalaches	3/225	1.3	0.3-3.9	3/200	1.5	0.3-4.3	0/13	0.0	0.0-24.7
13-Laval	3/231	1.3	0.3-3.8	2/179	1.1	0.1-4.0	0/12	0.0	0.0-26.5
14-Lanaudière	8/506	1.6	0.7-3.1	7/411	1.7	0.7-3.5	2/50	4.0	0.5-13.7
15-Laurentides	6/553	1.1	0.4-2.4	4/442	0.9	0.3-2.3	0/50	0.0	0.0-7.1
16-Montérégie	--	--	--	--	--	--	0/136	0.0	0.0-2.7
17-Centre-du-Québec	0/205	0.0	0.0-1.8	0/192	0.0	0.0-1.9	0/18	0.0	0.0-18.5

[1] All resident cats (n=720) and dogs (n=3,963) of the administrative region, whatever their travel history.

[2] Only the resident cats (n=692) and dogs (n=3,240) that did not travel out of their administrative region of residence within 14 days of tick collection.

[3] Cats and dogs not resident of the administrative region but that traveled in the region within 14 days of tick collection. Only the cats (n=15) and dogs (n=414) that traveled outside their administrative region of residence, but only to a single other region within 14 days of tick collection (excludes cats and dogs that traveled to two or more other administrative regions).

[4] Exact 95% confidence intervals.

Supplementary Table 7. Estimated risk of exposure to blacklegged ticks infected with *Babesia microti* in infested cats and dogs by host administrative region of residence and according to host travel history in Quebec, Canada from 2013-2017.

Animal species and administrative region	All resident[1]			Resident (no travel)[2]			Non-resident (travelers)[3]		
	Number exposed to infected ticks/total	Risk of exposure		Number exposed to infected ticks/total	Risk of exposure		Number exposed to infected ticks/total	Risk of exposure	
		%	CI (95%)[4]		%	CI (95%)[4]		%	CI (95%)[4]
**Cat**									
01-Bas-Saint-Laurent	0/65	0.0	0.0-5.5	0/64	0.0	0.0-5.6	0/3	0.0	0.0-70.8
02-Saguenay-Lac-Saint-Jean	0/41	0.0	0.0-8.6	0/41	0.0	0.0-8.6	0	--	--
03-Capitale-Nationale	0/119	0.0	0.0-3.1	0/111	0.0	0.0-3.3	0	--	--
04-Mauricie	0/82	0.0	0.0-4.4	0/81	0.0	0.0-4.5	0/1	0.0	0.0-97.5
05-Estrie	0/71	0.0	0.0-5.1	0/68	0.0	0.0-5.3	0	--	--
06-Montréal	0/246	0.0	0.0-1.5	0/226	0.0	0.0-1.6	0/2	0.0	0.0-84.2
07-Outaouais	0/61	0.0	0.0-5.9	0/59	0.0	0.0-6.1	0	--	--
08-Abitibi-Témiscamingue	0/21	0.0	0.0-16.1	0/20	0.0	0.0-16.8	0	--	--
09-Côte-Nord	0/4	0.0	0.0-60.2	0/4	0.0	0.0-60.2	0	--	--
10-Nord-du-Québec	0	--	--	0	--	--	0	--	--
11-Gaspésie-Îles-de-la-Madeleine	0/18	0.0	0.0-18.5	0/18	0.0	0.0-18.5	0	--	--
12-Chaudière-Appalaches	0/60	0.0	0.0-6.0	0/58	0.0	0.0-6.2	0/3	0.0	0.0-70.8
13-Laval	0/89	0.0	0.0-4.1	0/84	0.0	0.0-4.3	0	--	--
14-Lanaudière	0/153	0.0	0.0-2.4	0/150	0.0	0.0-2.4	0/4	0.0	0.0-60.2
15-Laurentides	1/152	0.7	0.0-3.6	0/145	0.0	0.0-2.5	0/5	0.0	0.0-52.2
16-Montérégie	--	--	--	--	--	--	0/14	0.0	0.0-23.2
17-Centre-du-Québec	0/57	0.0	0.0-6.3	0/56	0.0	0.0-6.4	0	--	--
**Dog**									
01-Bas-Saint-Laurent	0/173	0.0	0.0-2.1	0/152	0.0	0.0-2.4	0/24	0.0	0.0-14.3
02-Saguenay-Lac-Saint-Jean	0/240	0.0	0.0-1.5	0/220	0.0	0.0-1.7	0/15	0.0	0.0-21.8
03-Capitale-Nationale	0/742	0.0	0.0-0.5	0/607	0.0	0.0-0.6	0/33	0.0	0.0-10.6
04-Mauricie	1/528	0.2	0.0-1.1	1/462	0.2	0.0-1.2	0/49	0.0	0.0-7.3
05-Estrie	0/524	0.0	0.0-0.7	0/478	0.0	0.0-0.8	0/65	0.0	0.0-5.5
06-Montréal	4/1633	0.2	0.1-0.6	0/1018	0.0	0.0-0.4	0/53	0.0	0.0-6.7

Animal species and administrative region	All resident[1]			Resident (no travel)[2]			Non-resident (travelers)[3]		
	Number exposed to infected ticks/total	Risk of exposure		Number exposed to infected ticks/total	Risk of exposure		Number exposed to infected ticks/total	Risk of exposure	
		%	CI (95%)[4]		%	CI (95%)[4]		%	CI (95%)[4]
07-Outaouais	0/359	0.0	0.0-1.0	0/313	0.0	0.0-1.2	0/23	0.0	0.0-14.8
08-Abitibi-Témiscamingue	0/123	0.0	0.0-3.0	0/109	0.0	0.0-3.3	0/3	0.0	0.0-70.8
09-Côte-Nord	0/39	0.0	0.0-9.0	0/37	0.0	0.0-9.5	0/4	0.0	0.0-60.2
10-Nord-du-Québec	0/7	0.0	0.0-41.0	0/2	0.0	0.0-84.2	0/3	0.0	0.0-70.8
11-Gaspésie-Îles-de-la-Madeleine	0/81	0.0	0.0-4.5	0/76	0.0	0.0-4.7	0/2	0.0	0.0-84.2
12-Chaudière-Appalaches	0/381	0.0	0.0-1.0	0/329	0.0	0.0-1.1	0/38	0.0	0.0-9.3
13-Laval	0/483	0.0	0.0-0.8	0/363	0.0	0.0-1.0	0/23	0.0	0.0-14.8
14-Lanaudière	0/917	0.0	0.0-0.4	0/754	0.0	0.0-0.5	0/96	0.0	0.0-3.8
15-Laurentides	0/1090	0.0	0.0-0.3	0/878	0.0	0.0-0.4	0/134	0.0	0.0-2.7
16-Montérégie	--	--	--	--	--	--	1/292	0.3	0.0-1.9
17-Centre-du-Québec	0/477	0.0	0.0-0.8	0/436	0.0	0.0-0.8	0/37	0.0	0.0-9.5

[1] All resident cats (n=1,239) and dogs (n=7,797) of the administrative region, whatever their travel history.

[2] Only the resident cats (n=1,185) and dogs (n=6,234) that did not travel out of their administrative region of residence within 14 days of tick collection.

[3] Cats and dogs not resident of the administrative region but that traveled in the region within 14 days of tick collection. Only the cats (n=32) and dogs (n=894) that traveled outside their administrative region of residence, but only to a single other region within 14 days of tick collection (excludes cats and dogs that traveled to two or more other administrative regions).

[4] Exact 95% confidence intervals.

Supplementary Table 8. Descriptive statistics of the spatiotemporal clusters of exposure to blacklegged ticks infected with *Borrelia burgdorferi* in infested dogs in Quebec, Canada from 2010-2017.

Cluster ID	Pathogen	Number of dogs in cluster	Number (%) of dogs with infected ticks in cluster	Cluster time period	Cluster radius (km)	Relative risk	*p*-value
A	*Borrelia burgdorferi*	517	137 (26.5)	2011-2013	273	1.59	<0.001
B	*Borrelia burgdorferi*	10	9 (90.0)	2015	246	5.21	<0.01

Supplementary Table 9. Descriptive statistics of characteristics of submitted ticks, host species, month of collection and host travel history with *p*-value from exact univariable logistic regression modeling the exposure to blacklegged ticks infected with *Borrelia miyamotoi* in 4,683 infested animals in Quebec, Canada from 2014-2017.

Characteristics	Number of infested animals	Exposure to infected ticks		*p*-value[1]
		Number	%	
**Condition of submitted ticks[2]**				0.26
At least one alive	284	1	0.4	
All dead	4276	55	1.3	
**Quality of submitted ticks[2]**				0.38
At least one intact	3491	47	1.4	
All damaged	1069	9	0.8	
**Maximum level of engorgement of submitted ticks[2]**				0.79
Fully engorged	865	12	1.4	
Partially engorged	3631	45	1.2	
Not engorged	164	1	0.6	
**Number of tick(s) pooled for PCR testing**				0.40
1	4396	53	1.2	
2 or more	287	5	1.7	
**Month of tick collection**				0.002
January-March	18	2	11.1	
April-June	1235	23	1.9	
July-September	170	1	0.6	
October-December	3260	32	1.0	
**Host Animal**				0.99
Cat	720	9	1.3	
Dog	3963	49	1.2	
**Relative maximal travel distance for hosts within 14 days of tick collection[2]**				0.41
None / within municipality	3587	48	1.3	
Out of municipality	345	1	0.3	
Out of administrative region	504	6	1.2	
Out of province	188	2	1.1	

[1] Exact univariable logistic regression *p*-value

[2] Information was missing on condition and quality of ticks from 123 animals, level of engorgement for 23 animals and travel history for 59 animals.

Supplementary Table 10. Descriptive statistics of characteristics of submitted ticks, host species, month of collection and host travel history with *p*-value from exact univariable logistic regression modeling the exposure to blacklegged ticks infected with *Babesia microti* in 9,036 infested animals in Quebec, Canada from 2013-2017.

Characteristics	Number of infested animals	Exposure to infected ticks		*p*-value[1]
		Number	%	
**Condition of submitted ticks[2]**				0.99
At least one alive	384	0	0.00	
All dead	6871	4	0.06	
**Quality of submitted ticks[2]**				0.58
At least one intact	5545	4	0.07	
All damaged	1710	0	0.00	
**Maximum level of engorgement of submitted ticks[2]**				0.47
Fully engorged	1998	0	0.00	
Partially engorged	6447	6	0.09	
Not engorged	307	0	0.00	
**Number of tick(s) pooled for PCR testing**				<0.01
1	8573	3	0.03	
2 or more	463	3	0.65	
**Month of tick collection[3]**				
January-March	44	0	0.00	0.12
April-June	2919	3	0.10	
July-September	417	1	0.24	
October-December	5656	2	0.04	
**Host Animal**				0.59
Cat	1239	1	0.08	
Dog	7797	5	0.06	
**Relative maximal travel distance for hosts within 14 days of tick collection[4]**				<0.001
None / within municipality	6758	1	0.01	
Out of municipality	661	0	0.00	
Out of administrative region	1068	1	0.09	
Out of province	408	4	0.98	

[1] Exact univariable logistic regression *p*-value.
[2] Information on condition and quality of submitted ticks was missing for 1,781 animals, and on tick engorgement for 284 animals.
[3] For the variable 'Month of tick collection', the categories 'January-March' and 'October-December' were merged to avoid categories without any observations to permit model convergence.
[4] For the travel history, the categories 'Travel out of municipality' and 'Travel out of administrative region' were merged to avoid categories without any observations to permit model convergence. Information was missing for 141 animals.

Supplementary Table 11. Description and exact chi-square value of all possible combinations of cats and dogs coexposure to *Borrelia burgdorferi*, *Anaplasma phagocytophilum*, *Borrelia miyamotoi* and *Babesia microti* through the bites of infected blacklegged ticks in Quebec, Canada from 2010-2017.

	B. burgdorferi		***A. phagocytophilum***		***B. miyamotoi***		***B. microti***[1]	
	Number of pets infested with ticks	% of hosts exposed to positive ticks	Number of pets infested with ticks	% of hosts exposed to positive ticks	Number of pets infested with ticks	% of hosts exposed to positive ticks	Number of pets infested with ticks	% of hosts exposed to positive ticks
B. burgdorferi								
Positive	--	--	2379	3.6 [a]	1493	1.6	1762	0.23 [a]
Negative	--	--	10168	1.7 [b]	3190	1.1	7274	0.03 [b]
A. phagocytophilum								
Positive	253	33.6 [a]	--	--	119	4.2 [a]	184	0.00
Negative	12294	18.7 [b]	--	--	4564	1.2 [b]	8852	0.07
B. miyamotoi								
Positive	58	41.4	58	8.6 [a]	--	--	58	0.00
Negative	4625	31.8	4625	2.5 [b]	--	--	4623	0.09
B. microti								
Positive	6	66.7 [a]	6	0.0	4	0.0	--	--
Negative	9030	19.5 [b]	9030	2.0	4677	1.2	--	--

[1] Coexposures with this agent were only found in one cat and two dogs that traveled in the United Sates, and one dog that traveled to Ontario, Canada, within 14 days of tick collection.

[a,b] Percentages in pairwise comparisons with different superscript letters are statistically significantly different (p-value<0.05, Exact chi-square test)

2 Supplementary Figures

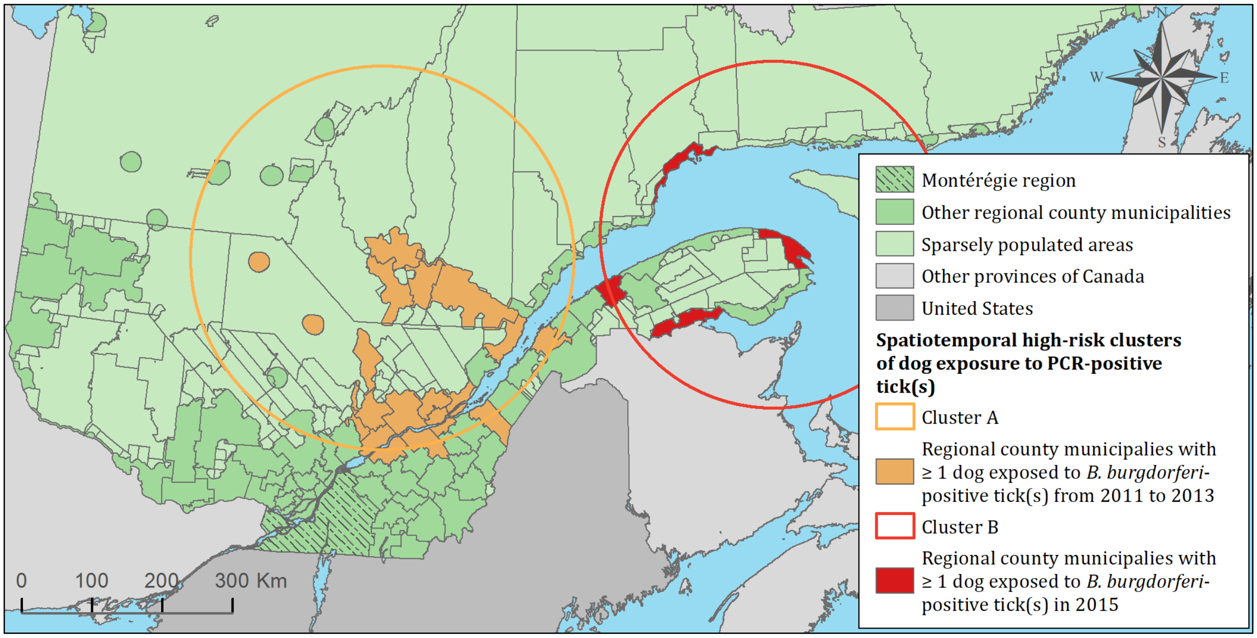

Supplementary Figure 1. Location of statistically significant spatiotemporal clusters of the 7,644 dogs exposed to *B. burgdorferi* infected blacklegged ticks. Only dogs that did not travel out of their municipality of residence within 14 days of tick collection were included. Cluster A: exposure to *Borrelia burgdorferi* from 2011 to 2013 inclusively. Cluster B: exposure to *Borrelia burgdorferi* in 2015. For each cluster, regional municipality counties with at least one *B. burgdorferi* infected tick detected during the cluster period. Sparsely populated areas represent unpopulated land or land with population density < 0.4 persons per km^2.
